# Evaluation of In Vitro and In Vivo Antiviral Activities of Vitamin D for SARS-CoV-2 and Variants

**DOI:** 10.3390/pharmaceutics15030925

**Published:** 2023-03-12

**Authors:** Chee-Keng Mok, Yan Ling Ng, Bintou Ahmadou Ahidjo, Zhen Qin Aw, Huixin Chen, Yi Hao Wong, Regina Ching Hua Lee, Marcus Wing Choy Loe, Jing Liu, Kai Sen Tan, Parveen Kaur, De Yun Wang, Erwei Hao, Xiaotao Hou, Yong Wah Tan, Jiagang Deng, Justin Jang Hann Chu

**Affiliations:** 1 Biosafety Level 3 Core Facility, Yong Loo Lin School of Medicine, National University of Singapore, Singapore 117599, Singapore; 2 Laboratory of Molecular RNA Virology and Antiviral Strategies, Department of Microbiology and Immunology, Yong Loo Lin School of Medicine, National University of Singapore, Singapore 117545, Singapore; 3 Infectious Disease Programme, Yong Loo Lin School of Medicine, National University of Singapore, Singapore 117597, Singapore; 4 Department of Otolaryngology, Yong Loo Lin School of Medicine, National University of Singapore, Singapore 119228, Singapore; 5 Guangxi Key Laboratory of Efficacy Study on Chinese Materia Medica, Guangxi University of Chinese Medicine, Nanning 530200, China; 6 Guangxi Collaborative Innovation Center for Research on Functional Ingredients of Agricultural Residues, Guangxi University of Chinese Medicine, Nanning 530200, China; 7 China-ASEAN Joint Laboratory for International Cooperation in Traditional Medicine Research, Guangxi University of Chinese Medicine, Nanning 530200, China; 8 Collaborative and Translation Unit for HFMD, Institute of Molecular and Cell Biology, Singapore 138673, Singapore

**Keywords:** SARS-CoV-2, calcitriol, vitamin D, drug screening

## Abstract

The COVID-19 pandemic has brought about unprecedented medical and healthcare challenges worldwide. With the continual emergence and spread of new COVID-19 variants, four drug compound libraries were interrogated for their antiviral activities against SARS-CoV-2. Here, we show that the drug screen has resulted in 121 promising anti-SARS-CoV-2 compounds, of which seven were further shortlisted for hit validation: citicoline, pravastatin sodium, tenofovir alafenamide, imatinib mesylate, calcitriol, dexlansoprazole, and prochlorperazine dimaleate. In particular, the active form of vitamin D, calcitriol, exhibits strong potency against SARS-CoV-2 on cell-based assays and is shown to work by modulating the vitamin D receptor pathway to increase antimicrobial peptide cathelicidin expression. However, the weight, survival rate, physiological conditions, histological scoring, and virus titre between SARS-CoV-2 infected K18-hACE2 mice pre-treated or post-treated with calcitriol were negligible, indicating that the differential effects of calcitriol may be due to differences in vitamin D metabolism in mice and warrants future investigation using other animal models.

## 1. Introduction

COVID-19, the disease caused by SARS-CoV-2 [1], was declared a pandemic by the World Health Organization (WHO) in March 2020 [2]. While vaccines are now available globally to provide acquired immunity to the disease, many drugs, such as remdesivir, are currently prescribed to manage the disease [3,4]. Despite the implementation of physical distancing, mask wearing, quarantine, and the tireless efforts expended for contact tracing, the rapid transmissibility of SARS-CoV-2 even during the asymptomatic phase has made containment of this virus extremely difficult. With increasing reports of new SARS-CoV-2 variants with higher virulence and reduced vaccine effectiveness, the global arms race to either discover, develop, or repurpose both old and new drugs continues at a breathtaking pace. To help identify promising therapeutics that may be repurposed against SARS-CoV-2 during this unprecedented time, we screened four compound libraries with the aim to narrow down potential drug candidates with antiviral activities against SARS-CoV-2. Out of the primary hits, we selected calcitriol, the active form of vitamin D, for further investigation on its antiviral role in SARS-CoV-2 infection through extensive cell-based and in vivo studies.

## 2. Materials and Methods

### 2.1. Cell lines and Viruses

African green monkey kidney cells (Vero E6; ATCC CRL-1586™), human hepatoma cells (HuH7; Dr Priscilla Yang, Harvard Medical School, USA), and human nasal epithelial cells (hNECs; Dr Wang De Yun, National University of Singapore, Singapore) were utilised in this study. Vero E6 and HuH7 cells were cultured in Dulbecco’s Modified Eagle’s Medium (DMEM) (Sigma-Aldrich, Burlington, MA, USA) supplemented with 10% heat-inactivated fetal calf serum (FCS) and buffered with 2 g sodium hydrogen carbonate.

hNECs were derived from in vitro differentiation of human nasal epithelial stem/progenitor cells (hNESPCs) obtained from healthy adult donors scheduled for septal plastic surgery. Approval to collect the tissue biopsies was obtained from the National Healthcare Group Domain-Specific Board of Singapore (DSRB Ref: D/11/228) and the institutional review board of the National University of Singapore (IRB Ref: 13-509). Written consent was also obtained from the donors prior to tissue biopsies collection. At the time of biopsies collection, all subjects were free of upper respiratory tract infections and rhinitis symptoms. Once the hNESPCs were isolated and enriched using standardised protocols [5,6], the hNESPCs were expanded and subjected to ALI culture in Transwells for in vitro differentiation [5,6]. The expanded hNESPCs were then transferred onto 12-well 0.4 μm Transwell inserts (Corning, Corning, NY, USA). Once the cells became confluent, growth medium was discarded and 700 μL of PneumaCult™-ALI Medium with inducer supplements (STEMCELL Technologies, Vancouver, BC, Canada) was added to the basal chamber to establish ALI conditions. The cells were cultured in ALI culture for 4 weeks, with a media change every 2–3 days. hNECs obtained after 3–4 weeks of differentiation were then subjected to SARS-CoV-2 infection. All cultures were maintained at 37 °C, 5% CO_2_.

Wild-type SARS-CoV-2 was isolated from a nasopharyngeal swab of a COVID-19 patient. The virus isolate was validated by qRT-PCR and propagated in Vero E6 cells with no more than 3 passages prior to drug screening. Alpha and beta variants of concern were isolated from COVID-19 patients and subsequently isolated and identified by qRT-PCR. All virus genomes have been released to GISAID by the National Public Health Laboratory, National Centre for Infectious Diseases, Singapore. The three variants used in this study include: Wild-type (lineage B, clade L, accession ID: EPI_ISL_574502), Alpha (lineage B.1.1.7, clade GRY, accession ID: EPI_ISL_754083), and Beta (lineage B.1.351.3, clade GH, accession ID: EPI_ISL_1173248). All virus work was performed in a Biosafety Level 3 (BSL-3) laboratory and all protocols were approved by the BSL-3 Biosafety Committee and Institutional Biosafety Committee of the National University of Singapore.

### 2.2. Preparation of Compound Libraries for Primary Screen

A primary screen to identify novel compounds with potential antiviral effects against SARS-CoV-2 infection was performed on four different libraries, namely a 462-compound ACE2-targeted compound library (CADD) (TargetMol, Boston, MA, USA), a 57-compound natural product library, a 500-compound flavonoids library (TimTec, Tampa, FL, USA), and a 1172-compound FDA-approved drug library (Selleckchem, Houston, TX, USA). Compounds were first dissolved in 100% dimethyl sulfoxide (DMSO) to a stock concentration of 10 mM, followed by dilution to 100 μM in serum-free media. Compounds were stored at −20 °C until further use.

### 2.3. Primary Screen with Pre-Treatment Using ACE2-Targeted Inhibitors and 57-Compound Natural Product Libraries

Vero E6 cells were seeded onto 96-well plates (Corning, Corning, NY, USA) at a seeding density of 1 × 10^4^ cells per 100 µL and incubated overnight prior to the drug inhibitory assay. Pre-infection treatment drug assays were performed by treating cells with 10 µM of ACE2-targeted inhibitors and the 57-compound natural product libraries for 2 h at 37 °C. A total of 0.1% DMSO and 100 μM remdesivir were included as vehicle and positive controls, respectively. Treated cells were then washed twice with phosphate-buffered saline (PBS) prior to SARS-CoV-2 infection at a multiplicity of infection (MOI) of 1 and incubated for 4 days at 37 °C, 5% CO_2_, before formalin fixation, analysis and hit selection.

### 2.4. Primary Screen with Post-Treatment Using FDA-Approved Drugs and Flavonoids Library

Vero E6 cells were seeded onto 96-well plates (Corning, Corning, NY, USA) at a seeding density of 1 × 10^4^ cells per 100 µL and incubated overnight. Cells were infected with SARS-CoV-2 infection at an MOI of 1 for 1 h. Final concentrations of 10 µM of FDA-approved drugs and 50 µM of flavonoids were added to SARS-CoV-2-infected cells, respectively. A total of 0.1% DMSO and 100 μM remdesivir were included as vehicle and positive controls, respectively. Cells were then incubated for 4 days at 37 °C, 5% CO_2_ before formalin fixation, analysis and hit selection.

### 2.5. Selection of Hits via Analysis of Cytopathic Effects (CPE)

For the selection of hits at 4 days post-infection, differences in cell viability caused by virus-induced CPE and/or compound-specific toxicity were visually analysed using light microscopy (Olympus, Shinjuku, Tokyo, Japan). Hits were identified and selected based on >50% reduction in CPE such as syncytium formation and rounding of cells in duplicate wells in comparison to the vehicle control.

### 2.6. Validation of Hits

Hits identified from the primary screens were validated using cell viability and dose-dependent inhibition assays on Vero E6 and HuH7 cells. To determine the cell viability profiles of the selected compounds, Vero E6 and HuH7 cells were seeded into 96-well plates at seeding densities of 1 × 10^4^ cells and 7.5 × 10^3^ cells per 100 µL, respectively. Both cell lines were pre-treated with the hit compounds from the ACE2-targeted library, namely citicoline, pravastatin sodium, and tenofovir alafenamide for 2 h at a range of concentrations (0.01, 0.1, 1, 10 and 20 µM). Hits identified from the FDA-approved drug library, namely imatinib mesylate, calcitriol, dexlansoprazole, and prochlorperazine dimaleate were added to the cells with the same concentration range and incubated for 4 days. After incubation, the media was removed from the plates and washed once with PBS before addition of alamarBlue Cell Viability Reagent (Thermo Fisher Scientific, Waltham, MA, USA) diluted 1:10 in media with 2% FCS. Fluorescence was detected after 2.5 h incubation at an excitation wavelength of 570 nm and an emission wavelength of 600 nm on an Infinite 200 Pro multiplate reader (Tecan, Männedorf, Zürich, Switzerland). Data obtained from compound-treated cells and vehicle-treated cells were normalised against those obtained from untreated cells.

Validation of the primary hits was performed via dose-dependent inhibition assays. Vero E6 and HuH7 cells were seeded onto 96-well plates and incubated overnight prior to drug inhibitory assays. Drug treatments were then divided into either a pre-infection treatment or a post-infection treatment.

- Pre-infection treatment with citicoline, pravastatin sodium, and tenofovir alafenamide at working concentrations of 0.01, 0.1, 1, 10, 20 µM was carried out for 2 h at 37 °C. The inhibitors were removed and washed twice with PBS prior to SARS-CoV-2 infection at an MOI of 1. After infection, unbound viruses were removed by washing twice with PBS and replaced with DMEM media.
- Post-infection treatment was performed for imatinib mesylate, calcitriol, dexlansoprazole, and prochlorperazine dimaleate. To this end, Vero E6 and HuH7 cells were first infected with SARS-CoV-2 at an MOI of 1 for 1 h at 37 °C and then incubated with the compound-containing DMEM media at working concentrations of 0.01, 0.1, 1, 10, 20 µM.

All dose-dependent inhibition assay plates were incubated for 4 days at 37 °C, 5% CO_2_, prior to the harvesting of viral supernatants for virus titration.

### 2.7. Validations of Imatinib Mesylate, Calcitrol and Citicoline with Primary Cell Line, hNEC at 10 µM

hNECs were seeded in apical chambers of the Transwell inserts for 4 weeks prior to drug inhibitory assays. Pre-infection treatment with citicoline at 10 µM was added to the basal chamber and carried out for 2 h at 37 °C. The inhibitors were removed and replaced with fresh PneumaCult™-ALI Medium (STEMCELL Technologies, Vancouver, BC, Canada) prior to SARS-CoV-2 infection at an MOI of 0.1. For validation of imatinib mesylate and calcitriol, hNECs were first infected with SARS-CoV-2 at an MOI of 0.1 for 1 h at 37 °C before incubation with the compounds at 10 µM as a post-infection treatment. All inhibition assay plates were incubated for 4 days at 37 °C, 5% CO_2_, prior to harvesting of viral supernatants for virus titration.

### 2.8. Plaque Assay

To determine the virus titre, viral supernatants harvested were 10-fold serially diluted in DMEM. A total of 200 µL of each serial diluted supernatant were applied to confluent Vero E6 cells. After 1 h of absorption, the inoculum was removed and 500 µL of 0.5% agarose overlay was added to each well and incubated for 3 days at 37 °C, 5% CO_2_ to facilitate plaque formation. The cells were fixed with formalin overnight and the agarose was removed before staining with crystal violet for 5 min. The number of plaques were counted and the virus titre of individual samples were expressed in the logarithm of plaque forming units (pfu) per mL.

### 2.9. Gene Expression Levels of Vitamin D Receptor (VDR), 24-Hydroxylase (24(OH)ase) and Anti-Microbial Protein Cathelicidin (LL-37) by Real-Time Quantitative Polymerase Chain Reaction (RT qPCR)

The relative messenger RNA (mRNA) expression of vitamin D receptor (VDR), 24 hydroxylase (24(OH)ase), and cathelicidin (LL-37) in Vero E6 and Huh7 cells were quantified by RT qPCR using the QuantStudio 6 real-time PCR system (Applied Biosystems, Waltham, MA, USA). To this end, two experiments were performed. In the first experiment, Vero E6 and Huh7 cells were either mock-infected with DMEM media, or SARS-CoV-2 infected at the MOI of 1 for 1 h. At 1 h post-infection, the cells were washed with PBS and then replaced with DMEM media and incubated for 6, 24 and 48 h at 37 °C to determine the baseline mRNA expression trends of each gene in uninfected or infected cells. In the second experiment, Vero E6 and Huh7 cells were infected with SARS-CoV-2 as above, and then treated with 0.01, 0.1, 1, 10 and 20 µM calcitriol and 0.1% DMSO as vehicle control at 1 h post-infection for 48 h. At each time point, total RNA was isolated using the RNeasy Mini Kit (Qiagen, Hilden, Germany). Following DNase digestion to remove unwanted DNA molecules, total RNA was subjected to RT qPCR using the SuperScript™ III One-Step RT-PCR System (Invitrogen, Waltham, MA, USA). Samples were assayed in 25 μL reaction mixtures containing 12.5 μL of 2X reaction mix, 10 μM of each probe, forward and reverse primers, 1 μg of template RNA, 2 μL of SuperScript™ III RT/Platinum™ *Taq* Mix and topped up to 25 μL using nuclease-free water. The sequences for the primers and probes for Vero E6′s VDR, 24(OH)ase and LL-37 were as follows: VDR (forward, 5′-CTTCAGGCGAAGCATGAAGC-3′; reverse, 5′-CCTTCATCATGCCGATGTCC-3′; probe, FAM 5′-AAGGCACTATTCACCTGCCCTTTCAA-3′ BHQ), 24(OH)ase (forward, 5′-CAAACCGTGGAAGGCCTATC-3′; reverse, 5′-CCAGCTTCATCACTTCCACT-3′, probe, FAM 5′-ATTACCGCAAAGAAGGCTACGGGCTG-3′ BHQ), and LL-37 (forward, 5′-TCACCGGAGGATTGTGACTTCAA-3′; reverse, 5′-TTAGGGTCACTGTTCCCACAC-3′; probe, FAM 5′-GAGGACGGGCTGGTGAAGCGG-3′ BHQ). The probe and primer sequences for Huh7′s VDR, 24(OH)ase and LL-37 were as follows: VDR (forward, 5′-CTTCAGGCGAAGCATGAAGC-3′; reverse, 5′-CCTTCATCATGCCGATGTCC-3′; probe, FAM 5′-AAGGCACTATTCACCTGCCCCTTCAA-3′ BHQ), 24(OH)ase (forward, 5′-CAAACCGTGGAAGGCCTATC-3′; reverse, 5′-CCAGCTTCATCACTTCCCCT-3′, probe, FAM 5′-ACTACCGCAAAGAAGGCTACGGGCTG-3′ BHQ), and LL-37 (forward, 5′-TCACCAGAGGATTGTGACTTCAA-3′; reverse, 5′-TGAGGGTCACTGTCCCCATAC-3′; probe, FAM 5′-AAGGACGGGCTGGTGAAGCGG-3′ BHQ). Reaction mixtures were subjected to a reverse transcription at 45 °C for 30 min, a polymerase activation at 94 °C for 2 min, followed by 40 cycles of denaturation, annealing, and extension at 94 °C for 15 s, 60 °C for 20 s, and 68 °C for 15 s, respectively. The results obtained were in the form of threshold cycles (C_T_ values), and the data were normalized to the endogenous housekeeping gene beta-actin using the 2^−ΔΔCT^ method [7]. In cases where the C_T_ values were undetermined, a C_T_ value of 40 is assigned to calculate the expression ratio of the gene of interest.

### 2.10. Protein Expression and Analysis

#### 2.10.1. Protein Extraction

To evaluate the protein expression levels of SARS-CoV-2 and VDR in Vero E6 cells treated or untreated with calcitriol post-infection, Vero E6 wells were either mock-infected with DMEM media or infected with SARS-CoV-2 at MOI-1 for one hour. After one hour of incubation, the cells were washed with PBS and then replaced with DMEM containing either 0.1% DMSO as a vehicle control, or 20 µM calcitriol and incubated for 6, 24 and 48 h. At each indicated time point, total cellular proteins from Vero E6 cells were harvested using 1% NP-40 lysis buffer. All samples were boiled at 65 °C for 10 min and stored at −20 °C.

#### 2.10.2. Sodium Dodecyl Sulfate-Polyacrylamide Gel Electrophoresis (SDS-PAGE) and Western Blot Analysis

To run SDS-PAGE, all frozen protein samples were reheated at 100 °C for 10 min before being loaded into a precast polyacrylamide gel consisting of 10% resolving gel and 5% stacking gel. The protein samples were electrophoresed using the mini gel apparatus (Bio-Rad, Hercules, CA, USA) at a constant voltage of 120 V until the dye marker had run to the bottom of the gel. Post SDS-PAGE, the proteins were transferred to the PDVF membrane by semi-dry transfer (Bio-Rad, Hercules, CA, USA). The membranes were blocked for 1 h in blocking buffer (5% BSA in TBST buffer) before incubating with primary antibodies [anti-VDR (Proteintech, Hubei, China, cat no. 16922-1-AP), anti-SARS-CoV-2 Spike (Sino-Biologicals, Beijing, China, cat no. 40592-T62)], and loading control anti-beta-actin for 1 h at room temperature. After washing five times with TBST at 5 min intervals, the membrane was incubated with 1:10,000 diluted anti-rabbit or anti-mouse IgG antibodies conjugated with horseradish peroxidase in blocking buffer for 1 h at room temperature. After washing five times with TBST, the polypeptides were detected with a chemiluminescence kit (Millipore, Burlington, MA, USA) according to the manufacturer’s protocol before loading onto a chemiluminescent western blot scanner (Li-Cor, Lincoln, NE, USA). The developed western blot images were saved as TIFF images, and the intensity of the protein bands was determined semi-quantitatively via ImageJ Version 1.51 (National Institute of Health, Bethesda, MD, USA). The band intensities were normalized with beta-actin as the loading control.

### 2.11. Animals and Treatment Regimen

Eight-week-old male and female K18-hACE2 transgenic female mice (InVivos Pte Ltd., Singapore) were used for this study. The mice were housed in an ABSL-3 facility for 72 h for acclimatization prior to the start of the experiment. Intraperitoneal injection and oral gavage were used as treatment delivery methods. The mice were subjected to a three-day and seven-day pre-treatment regime with a calcitriol concentration of 5 and 0.5 μg/kg prior to infection for intraperitoneal injection and oral gavage respectively. Post-treatment was carried out for an additional four days after infection. The mice were inoculated intranasally with 10^3^ PFU of SARS-CoV-2 virus suspension in filtered PBS. Baseline body weights were measured prior to treatment and infection. Body weight, survival, and physiological conditions were monitored by two personnel daily post-infection for the duration of the experiment. Mice were segregated into two identical groups for each treatment condition for each of the three variants, the first group for observational studies for body weight, physiological changes, and survival; the second group for the assessment of viral titre at 4 days post-infection (dpi) and histological analyses. To assess viral titre, mice from each group were sacrificed 4 dpi with the spleen, liver, brain, and lungs harvested and halved for plaque assay and histological examination. Harvested organs were homogenized in 500 μL DMEM (Cytiva, South Logan, UT, USA) supplemented with an antibiotic and antimycotic (Gibco, Waltham, MA, USA) and titrated in Vero E6 cells using plaque assays. All animal experiments were conducted in a Biosafety Level 3 (BSL-3) facility in accordance with the National University of Singapore (NUS) Institutional Animal Care and Use Committee (IACUC) protocol number R20-0504, and the NUS Institutional Biosafety Committee (IBC) and NUS Medicine BSL-3 Biosafety Committee (BBC) approved SOPs.

### 2.12. Statistical Analyses

Results were analyzed using GraphPad Prism version 8 (GraphPad Software, San Diego, CA, USA). Data are reported as means ± standard deviation (SD). One-way analyses of variance (ANOVA) were used to evaluate the statistical significance of data obtained. Drug-treated samples expressing a statistical difference when compared to control samples were subsequently subjected to a Dunnett’s post-test, with * denoting that *p* < 0.05, ** denoting that *p* < 0.01 and *** denoting that *p* < 0.001. A paired Student’s T-test was also performed to evaluate the statistical significance of the mRNA expression levels between mock- and SARS-CoV-2 infected samples.

## 3. Results

### 3.1. Cytopathic Effect (CPE) Based Screening of Compound Libraries

**Figure 1:**
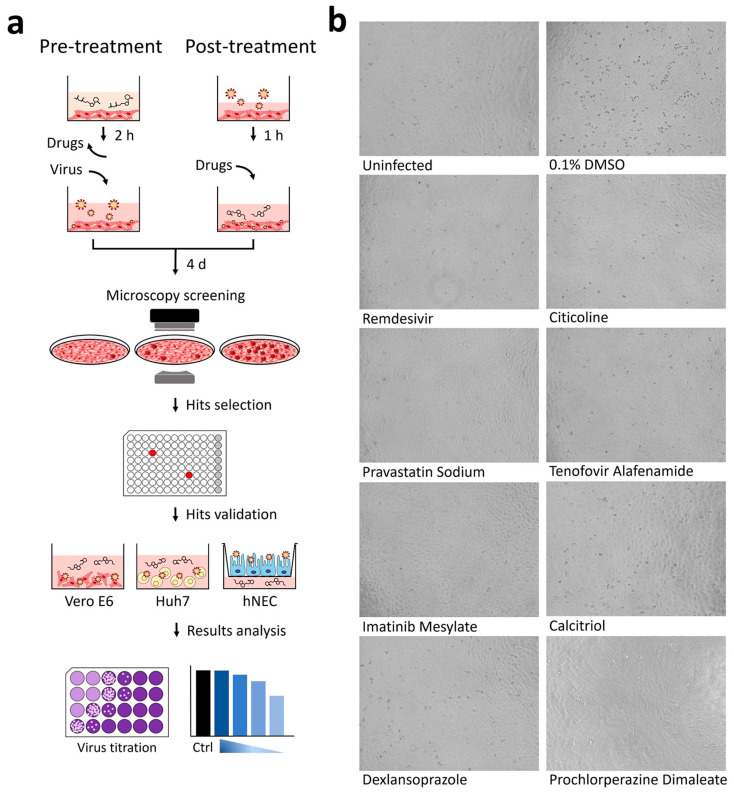
Schematic overview of the anti-SARS-CoV-2 drug screening procedure. (**a**) ACE2 targeted inhibitors and compounds from the natural product library were applied prior to infection with SARS-CoV-2 (pre-infection treatment) while compounds from the FDA-approved library and the flavonoid library were applied post infection (post- infection treatment). Out of 2191 compounds tested, 121 displayed viral CPE reductions and 7 were selected for further validations using Vero E6 and HuH7 cell lines and hNEC. (**b**) Uninfected cells, infected cells treated with 0.1% DMSO, and infected cells treated with remdesivir were included as controls in the screen. Following treatment with citicoline, pravastatin sodium, tenofovir alafenamide, imatinib mesylate, calcitriol, dexlansoprazole, and prochlorperazine dimaleate, reduced CPE was observed when compared to the DMSO control.

In an effort to identify potential candidates for SARS-CoV-2 chemoprophylaxis, we performed a virus-induced cytopathic effect (CPE) based screen of several small molecule libraries in SARS-CoV-2-infected Vero E6 cells (Figure 1a). The African green monkey kidney epithelial Vero E6 cells were used for the screen as these cells are highly susceptible to coronaviruses and exhibit obvious CPE upon infection. A 57-compound natural product library and a library of 462 ACE2 targeted inhibitors (the ACE2 receptor was identified to be necessary for SARS-CoV-2 infection [8]) were used in a pre-infection treatment screen to identify potential viral entry inhibitors, while a post-infection treatment screen was performed using both a 500 compound flavonoid library and a 1172 FDA-approved compound library in order to identify potential inhibitors targeting the post-entry steps of the SARS-CoV-2 replication cycle. For the pre-infection treatment screen, Vero E6 cells were treated with compounds for two hours. After which, the treated cells were then washed twice with PBS prior to infection with SARS-CoV-2. After 1 h of infection, the cells were then washed again with PBS and incubated with DMEM. The post-infection treatment screen on the other hand was performed by adding compounds to the Vero E6 cells 1 h post-infection with SARS-CoV-2. Compounds that showed less than 50% CPE compared to the 0.1% DMSO vehicle control with SARS-CoV-2 infection were identified as hits (Tables S1–S3). Using this method, we identified 31 compounds from the pre-infection treatment screen and 90 compounds from the post-infection treatment screen with activity against SARS-CoV-2 (Table 1). As expected, our hit list included the tyrosine kinase inhibitors masitinib and imatinib mesylate [9], the antiretroviral drug lopinavir [10], and the calpain inhibitor calpeptin [11]–all compounds reported to inhibit SARS-CoV, SARS-CoV-2 or MERS-CoV. This provides the robustness and confidence of our primary screen for potential antivirals against SARS-CoV-2.

### 3.2. Validation of Hit Compounds via Dose-Dependent and Cell Viability Assays

Out of these primary hits, seven compounds were selected for downstream validation (Table 2). These included three compounds from the pre-infection treatment screen (citicoline, pravastatin sodium and tenofovir alafenamide) and four compounds from the post-infection treatment screen (imatinib mesylate, calcitriol, dexlansoprazole, and prochlorperazine dimaleate). These compounds were selected based on the level of CPE inhibition in the primary screens (Figure 1b), known mechanisms of action, and existing FDA approval or Generally Recognized as Safe (GRAS) status. FDA approval was considered an important factor as pre-existing data on safety and dosage would allow expedited decisions to be made regarding the potential use of these compounds in vulnerable populations to stymie the current pandemic. 

**Figure 2:**
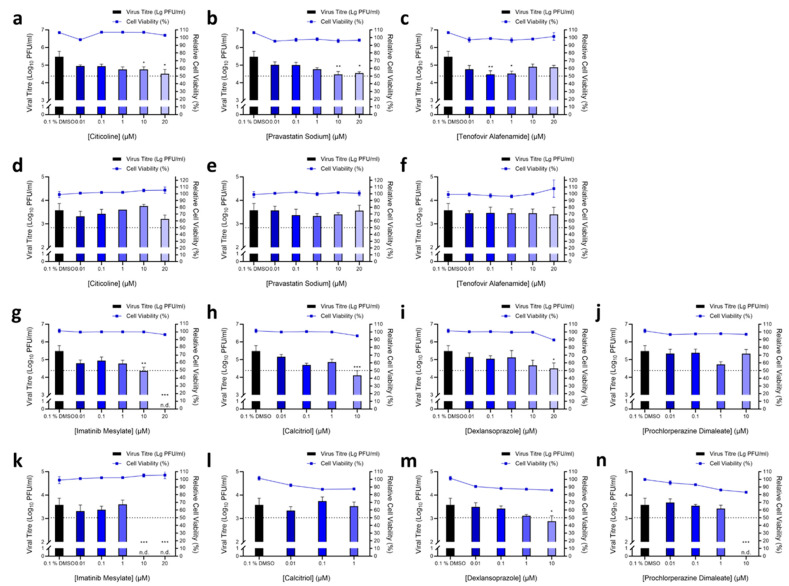
Validation of primary hits. For validating compounds with activity pre-infection, Vero E6 cells were first pre-treated with increasing concentrations of (**a**) citicoline, (**b**) pravastatin sodium and (**c**) tenofovir alafenamide prior to infection with SARS-CoV-2. Similarly, Huh7 cells were pre-treated with (**d**) citicoline, (**e**) pravastatin sodium and (**f**) tenofovir alafenamide and subsequently infected with SARS-CoV-2. For post-infection treatment validation, Vero E6 cells were infected with SARS-CoV-2 and treated with increasing concentrations of (**g**) imatinib mesylate, (**h**) calcitriol, (**i**) dexlansoprazole and (**j**) prochlorperazine dimaleate. Similarly, HuH7 cells were also infected then treated with a range of concentrations of (**k**) imatinib mesylate, (**l**) calcitriol, (**m**) dexlansoprazole and (**n**) prochlorperazine dimaleate. The primary and secondary exes correspond to the viral titre and relative cell viability, and the dashed line represents the CC_50_ cut-off for cell viability. One-way ANOVA and Dunnett’s post-test were used to determine statistical differences, with * denoting *p* < 0.05, ** denoting *p* < 0.01 and *** denoting *p* < 0.001. Error bars represent the standard deviation observed from the means of triplicates performed for both cell viability and dose-dependent inhibition studies.

Validation assays to determine changes in infectious virus titres upon treatment were carried out by testing selected hit compounds in dose-dependent assays in Vero E6 to confirm the primary screen observation and also in the human hepatocarcinoma HuH7 cell line as the latter cell line expresses high levels of the ACE2 receptor [12] and supports replication of coronaviruses [13]. Cell viability assays were also carried out to ensure that the reduction of SARS-CoV-2 titres was not due to the cytotoxic effects of the compounds on host cells. CC_50_ and IC_50_ were obtained for each of the seven compounds in Vero E6 cells and HuH7 cells (Table 3), and where possible selectivity index values were calculated. Pre-infection treatment of Vero E6 cells with citicoline and pravastatin sodium resulted in the dose-dependent inhibition of SARS-CoV-2 (Figure 2a,b), while a non-dose-dependent inhibition of SARS-CoV-2 at lower concentrations was observed with tenofovir alafenamide (Figure 2c). These observations were however not recapitulated in the HuH7 cell line (Figure 2d–f). Post-infection treatment with ≥10 μM imatinib mesylate resulted in a reduction in SARS-CoV-2 titres to non-detectable levels in both Vero E6 and HuH7 cells (Figure 2g,k), and a similar finding was observed with ≥10 μM prochlorperazine dimaleate in HuH7 cells (Figure 2n). Significant reductions of at least 0.4 log_10_ of SARS-CoV-2 were also noted upon treatment with ≥10 μM dexlansoprazole (Figure 2i,m) and post-infection treatment with 10 μM calcitriol resulted in a 1.3 log_10_ reduction of SARS-CoV-2 titres in Vero E6 cells (Figure 2h). This finding could not be recapitulated in HuH7 cells, (Figure 2l) most likely because the CC_50_ value of calcitriol was 4.7 μM in HuH7 cells (Table 3).

**Figure 3:**
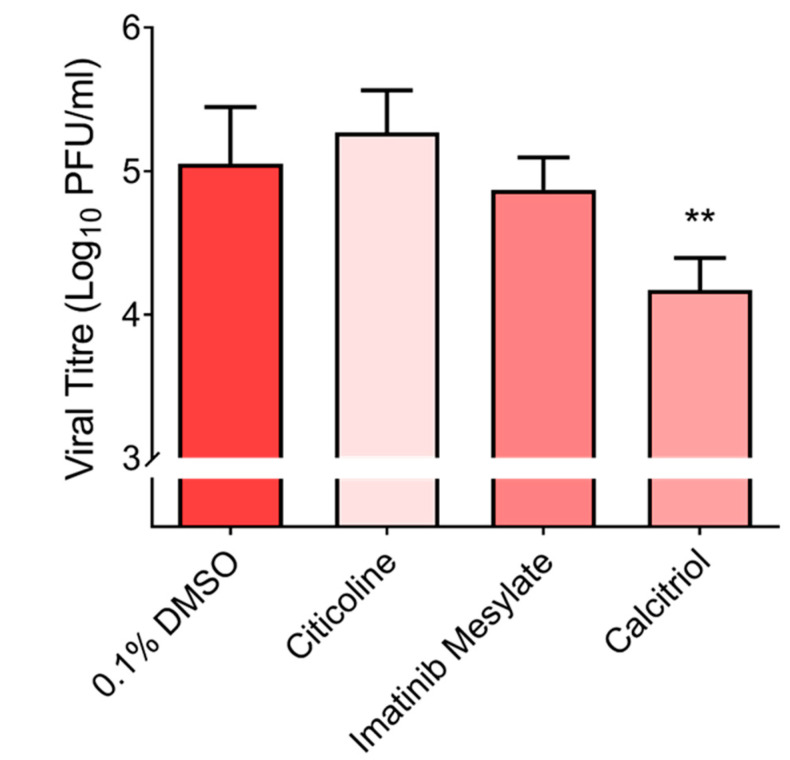
Validation of primary hits in hNECs. Selected primary hits were further validated in hNECs. Cells were either pre-treated with 10 μM citicoline prior to SARS-CoV-2 infection or treated following SARS-CoV-2 infection with 10 μM imatinib mesylate and 10 μM calcitriol. One-way ANOVA and Dunnett’s post-test were used to determine statistical differences, with ** denoting that *p* < 0.01. Error bars represent the standard deviation observed from the means of triplicates performed for dose-dependent inhibition studies.

Given that the HuH7 cell line is a hepatocarcinoma cell line and therefore not the first point of entry for SARS-CoV-2 in humans, we decided to test the three most promising compounds (imatinib mesylate, citicoline, and calcitriol) against SARS-CoV-2 in the primary human nasal epithelial cell line (hNEC) that is a known in vivo target of SARS-CoV-2 [14] (Figure 1a). Despite its significant activity in the continuous cell lines (Vero E6 and HuH7), in hNECs, imatinib mesylate only displayed a 0.2 log_10_ reduction in viral titre (Figure 3). Interestingly, out of the three compounds, only calcitriol proved effective against SARS-CoV-2 with a reduction of 0.69 log_10_ in viral titre (Figure 3).

### 3.3. Elucidating the Antiviral Role of Calcitriol by RT qPCR

Several clinical studies have suggested a negative correlation between serum vitamin D levels with the morbidity and mortality of COVID-19 cases [15,16]. Given that calcitriol acts primarily as a ligand of the nuclear vitamin D receptor (VDR), we hypothesize that SARS-CoV-2 may modulate the host antiviral immune response via the VDR pathway. Under normal physiological circumstances, calcitriol, being the biologically active form of vitamin D, binds to VDR to form a heterodimer with retinoid-X receptor (RXR) [17]. The calcitriol-VDR-RXR complex would then migrate to the nucleus to bind the vitamin D response element (VDRE) in the promoter region and trigger the recruitment of transcriptional co-activators and co-repressors to modulate a diverse range of genes [17]. Some of these genes regulated by the VDR pathway include the antimicrobial peptide cathelicidin (LL-37), a known selective inhibitor of cathepsin L [18] involved in SARS-CoV virus membrane fusion and entry [19,20], and 24-hydroxylase (24(OH)ase), which helps to metabolize calcitriol through a series of hydroxylation/oxidation reactions [21].

**Figure 4:**
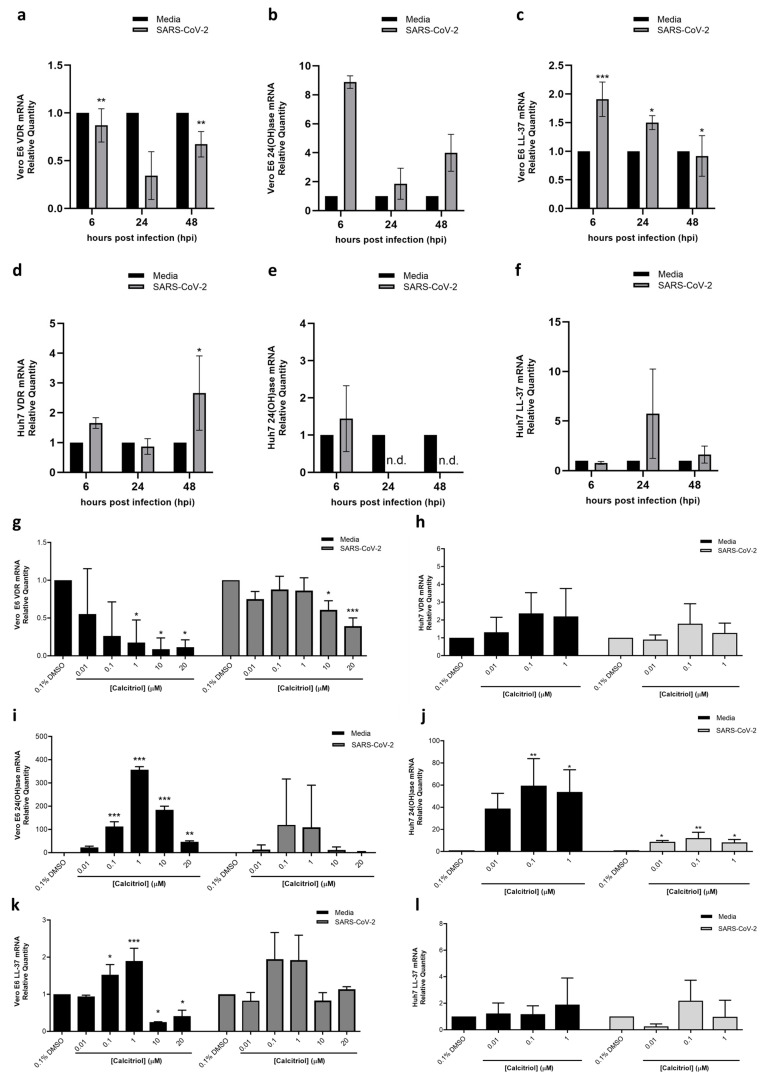
SARS-CoV-2 and calcitriol modulate vitamin D Receptor (VDR), 24-hydroxylase (24(OH)ase), and cathelicidin (LL-37) in Vero E6 and Huh7 cells. In the first part of the experiment, Vero E6 and Huh7 cells were either mock-infected with media, or SARS-CoV-2 infected and harvested at indicated timepoints in the absence of calcitriol for baseline VDR, 24(OH)ase, and LL-37 mRNA expression. Vero E6 expression of (**a**) VDR, (**b**) 24(OH)ase and (**c**) LL-37 and Huh7 expression of (**d**) VDR, (**e**) 24(OH)ase and (**f**) LL-37 were as shown. In the second part of the experiment, Vero E6 and Huh7 cells were either mock-infected with media or SARS-CoV-2 for 1 h as above, then treated with 0.01, 0.1, 1, 10 or 20 µM calcitriol following infection. At 48 h post infection, the cells were harvested to check for VDR, 24(OH)ase, and LL-37 mRNA expression. Vero E6 mRNA expression of (**g**) VDR, (**i**) 24(OH)ase and (**k**) LL-37 and Huh7 expression of (**h**) VDR, (**j**) 24(OH)ase and (**l**) LL-37 were as shown. Data are represented as relative quantity to 0.1% DMSO control, or as relative quantity to mock-infected control. One-way ANOVA and Dunnett’s post-test and paired *t*-test were used to determine statistical differences, with * denoting *p* < 0.05, ** denoting *p* < 0.01 and *** denoting *p* < 0.001. Error bars represent the standard deviation observed from the means of triplicates performed for mRNA expression studies.

To this end, two experiments were conducted to determine the role of calcitriol in the VDR pathway during virus infection. In the first experiment, Vero E6 and Huh7 cells were either mock infected with media or infected with SARS-CoV-2 and harvested at 6-, 24- and 48-h post-infection (hpi) to determine the baseline mRNA expression levels of VDR, 24(OH)ase, and LL-37 without calcitriol treatment. Here, we show that Vero E6 cells constitutively express VDR, 24(OH)ase, and LL-37 in mock-infected samples across all timepoints (Figure 4a–c). When Vero E6 cells were infected with SARS-CoV-2, mRNA expression of VDR and LL-37 were significantly reduced with time (Figure 4a,c). While 24(OH)ase expression was on average five-fold higher than mock-infected Vero E6 cells across all timepoints, its expression was also reduced to two- and four-fold overexpression relative to mock-infected cells at 24- and 48-hpi following infection, respectively (Figure 4b). On the other hand, in Huh7 cells, there is a slight difference in the baseline expression of VDR, 24(OH)ase, and LL-37 compared to Vero E6 cells. In Huh7 cells, while VDR and LL-37 expression were constitutively expressed from 6 to 48 hpi (Figure 4d,f), 24(OH)ase was undetectable in all timepoints regardless of whether the cells are infected or not, except in SARS-CoV-2 infected cells at 6 hpi (Figure 4e). This may be due to species differences in 24(OH)ase activity [21], and suggests that in human cell lines such as Huh7, 24(OH)ase is usually not expressed in the absence of calcitriol since its function is to metabolize calcitriol into calcitroic acid. VDR expression in Huh7 cells also increased with time in SARS-CoV-2 infected cells compared to mock-infected cells-a contrast to Vero E6, which reduces VDR expression with infection time (Figure 4a,d). Huh7 LL-37 expression is also comparable to mock-infected cells in the absence of calcitriol, except at 24 hpi (Figure 4f).

Knowing the baseline expression trends of VDR, 24(OH)ase, and LL-37 in Vero E6 and Huh7 cells, we next investigate the mRNA expression levels of the same three genes in the presence of calcitriol in the second part of the experiment. Vero E6 and Huh7 cells were both either mock-infected with media or infected with SARS-CoV-2 as above, and then subsequently treated with 0.01, 0.1, 1, 10 and 20 µM calcitriol along with 0.1% DMSO as a vehicle control for 48 h. Similarly, 0.01, 0.1 and 1 µM calcitriol were used in Huh7 cells as the CC_50_ of calcitriol in Huh7 cells is 4.7 µM. The results show that with calcitriol treatment, Vero E6 VDR expression is reduced in a dose-dependent manner in both mock- and SARS-CoV-2 infected cells, with its reduction being most significant at 20 µM (Figure 4g). Conversely, 24(OH)ase and LL-37 in calcitriol-treated Vero E6 cells showed an increase in expression in a dose-dependent manner up to 1µM (Figure 4i,k). Compared to calcitriol untreated Vero E6 cells at 48hpi, calcitriol upregulates LL-37 expression, which serves a critical role in the innate immune defense against infections (Figure 4c,k). On the other hand, Huh7 cells showed an increase in VDR expression with increasing concentrations of calcitriol (Figure 4h). Despite differences in VDR expression between the two cell lines, 24(OH)ase and LL-37 both increased their expression with calcitriol treatment (Figure 4j,l).

**Figure 5:**
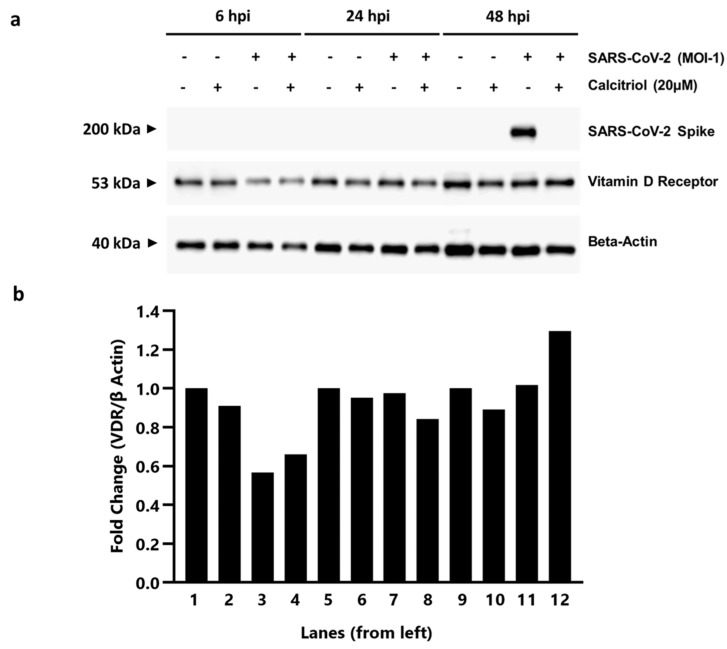
Western blot analysis of SARS-CoV-2 spike and VDR proteins in Vero E6 cells. (**a**) Vero E6 cells are either mock infected with media (−) or with SARS-CoV-2 (+) for 1 h. After 1 h post-infection, the cells are then either untreated (−) or treated (+) with 20 µM calcitriol for 6-, 24- and 48- hours before harvesting of total cell lysate. (**b**) Densitometry quantitation of protein expression levels is shown as fold changes to beta-actin. Full-length blots are shown in Supplementary Figure S1.

To understand if the mRNA expression trends of VDR, 24(OH)ase, and LL-37 observed in both cell lines were translated at the protein level, a Western blot was performed using Vero E6 cells. To this end, Vero E6 cells were either mock-infected with media or SARS-CoV-2 as above and were either treated with or without 20 µM calcitriol after infection. Total cell lysates were harvested at 6-, 24- and 48 hpi for the detection of SARS-CoV-2 and VDR only, as many commercial antibodies have been unsuccessful at detecting Vero E6′s 24(OH)ase and LL-37 proteins. Here, we show that without SARS-CoV-2 infection, VDR expression level would reduce by 10% when treated with calcitriol (lane 1 and 2 for 6 hpi, lanes 5 and 6 for 24 hpi, and lanes 9 and 10 for 48 hpi) (Figure 5a,b). However, during early SARS-CoV-2 infection at 6 hpi, there is over 40% reduction in VDR protein compared to its mock-infected counterparts (lanes 1 and 3). These observations coincide with the RT-qPCR data in Figure 4a, where SARS-CoV-2 infection would reduce VDR expression in absence of calcitriol. At 48 hpi, SARS-CoV-2 spike protein can be detected in SARS-CoV-2 infected, calcitriol untreated lysate (Figure 5a, lane 11), while the infected cells treated with 20 µM calcitriol did not have any SARS-CoV-2 spike protein (Figure 5a, lane 12). This corroborates our earlier cell-based studies that calcitriol is effective in inhibiting virus production in Vero E6 cells.

### 3.4. In Vivo Study of Calcitriol in K18-hACE2 Mice 

**Figure 6:**
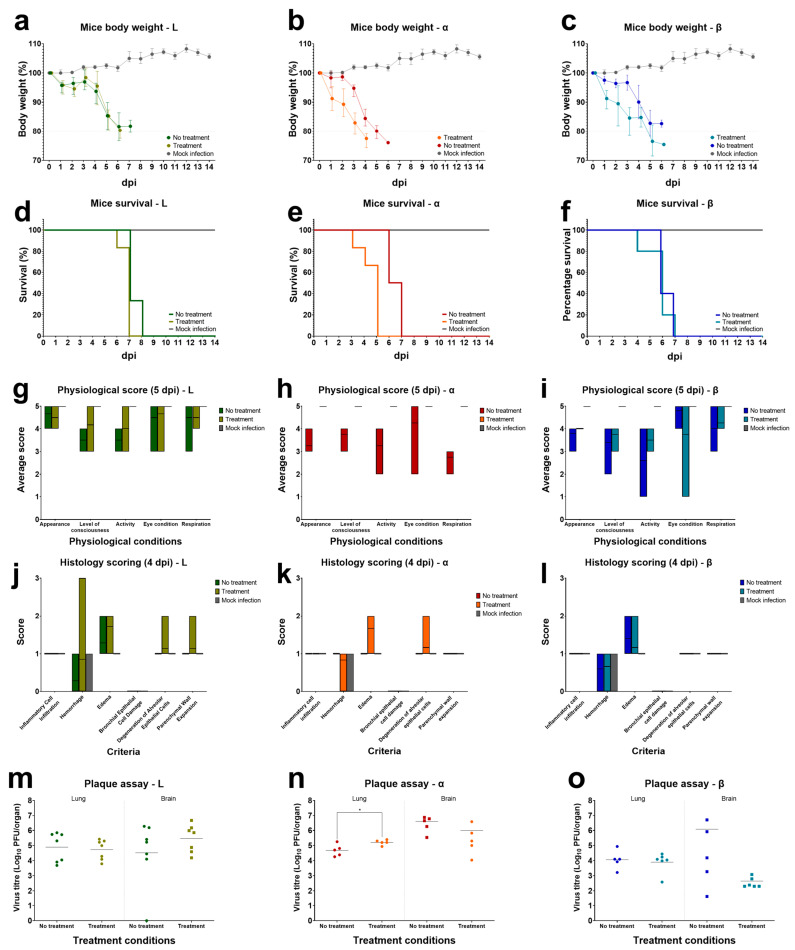
In vivo study of calcitriol treatment. 8-week old K18-hACE2 male and female mice were separated into treatment and non-treatment groups. Treatment study group has been given 3 days pre-treatment and 5 days post-treatment of calcitriol (5 μg/kg) via intraperitoneal injection. The non-treatment group has been given a PBS as a treatment control. Next, 10^3^ PFU of SARS-CoV-2 (L, Alpha, and Beta variants) were inoculated through intranasal delivery to all groups after pre-treatment. Physiological parameters: body weight (**a**–**c**), survival rate (**d**–**f**), and physiological conditions were monitored after infections. For comparison between individual physiological conditions, 5 dpi infected mice were compared between treatment groups of all three variant infected mice (**g**–**i**). Scoring of physiological conditions was based on five criteria: appearance of the mouse coat, level of consciousness, activity level, eye condition, and respiratory quality. To compare the severity of damage in mouse tissues after infection, left lung lobes from 4 dpi mice were processed for histological analyses and scored based on six criteria to determine severity (**j**–**l**). The six criteria are inflammatory cell infiltration, haemorrhage, oedema, bronchial epithelial cell damage, degeneration of alveolar epithelial cells, and parenchymal wall expansion. For viral load determination, right lung, brain, liver, and spleen tissues from the same 4 dpi mice were harvested and homogenised for virus titration (**m**–**o**). Data is not shown for liver and spleen tissues. Statistical significance is determined with a two-tailed unpaired *t*-test, with * denoting *p* < 0.05. Survival group: variant L: n = 6 (treatment and no treatment); Alpha: n = 6 (treatment), n = 4 (no treatment); Beta: n = 6 (treatment), n = 5 (no treatment). 4 dpi group: variant L: n = 7 (treatment and no treatment); Alpha and Beta: n = 5 each (treatment and no treatment). Mock infection, n = 3.

Calcitriol is known to induce and regulate vitamin D receptor signalling in mice [22,23,24]. Hence, to validate the effectiveness of calcitriol in vivo, 8-week old K18-hACE2 transgenic female mice were given calcitriol as a prophylactic treatment through a low-dosage oral gavage regiment [25,26] at a dose of 0.5 µg/kg 7 days prior to infection. Post-treatment with calcitriol was given daily until 4 dpi. We observed no significant effect on the loss of body weight, survival rate of the mice, and the physiological state of the mice (Figure S4a–c). Titration of viral load in both brain and lung tissues among the study group also did not show any significant differences (Figure S4g). The results suggested the possibility of the lack of efficacy through low-dosage delivery of calcitriol. The experimental setup was subsequently modified to increase the dosage of calcitriol at 5 µg/kg, delivered through intraperitoneal injection [27]. Mice were subjected to the treatment regime 3 days prior to infection, post-treatment of calcitriol was given daily till 4 dpi. Weight changes (Figure 6a–c) and physiological scores (Figure 6b) were monitored daily until the experimental end point at 14 dpi or until death occurred. No significant changes in body weight were observed for both treatment and non-treatment groups in the initial four days post-infection for all variants, with body weight losses observed to be less severe for both Alpha- and Beta-variant infected mice. For variant L, both treatment and non-treatment groups experienced a 10% weight loss subsequently on 5 dpi and stabilised at around 80% of initial body weight before succumbing to the virus infection. Alpha- and Beta-variant infected mice followed a similar decrease in body weight as variant L, however with an earlier reduction in body weight for both treatment groups. The non-treatment groups were observed to have a similar loss of body weight for all three variant infected mice. Survival rates between both treatment groups were similar for all three variants, with all mice succumbing at 6 and 7 dpi (treatment and no treatment; L), 4 and 6 dpi (Alpha), 6 dpi (Beta). Interestingly, all mice without calcitriol treatment were able to survive one to two days longer than the treatment group.

Physiological scoring of the infected mice was evaluated based on five criteria: the appearance of the mouse coat, level of consciousness, activity level, eye condition, and respiration quality. These conditions were scored on a scale of 1 to 5; 5 being normal physiological state and 1 being the most severe (Figure S2a–c). Despite the similar changes in the weight loss and survival rate for both study groups, we observed the physiological state of the treatment group to be better and less varied on average. We selected a comparison point at 5 dpi for a breakdown of the five different conditions among the treatment groups in the variants infected mice (Figure 6g–i). Physiological scoring for all five conditions for variant L was observed to be less severe for the treatment group in comparison to the non-treatment group. This trend was observed similarly in the Beta variant group for coat appearance, level of consciousness, activity, and respiration. However, eye condition was noticeably more variable and more severe on average for the treatment group. For the Alpha variant group, all treatment mice reached their end point on 4 dpi, with the severity of the five conditions similar in comparison to the other two non-treatment groups with L and Beta variants.

Harvested left lung tissues from the 4 dpi groups were processed for histology staining and scored on a scale of 0 to 3; 0 being the normal tissue state and 3 being severely inflamed. Six criteria were selected for the scoring of the tissues: inflammatory cell infiltration, haemorrhage, oedema, bronchial epithelial cell damage, degeneration of alveolar epithelial cells, and parenchymal wall expansion (Figure 6j–l). Tissues for the mock treatment group were scored at 1, indicating a mild level of inflammation. For the variant L infected group, we observed a slightly more severe histological scoring for haemorrhage, oedema, degeneration of alveolar epithelial cells, and parachymal wall expansion for the treatment group in comparison to the non-treatment group. The same observation was noted with both Alpha and Beta variants for haemorrhage and oedema. For inflammatory cell filtration, the severity was similar across treatments among the three variants.

Viral loads were determined through titration from tissues harvested from the 4 dpi groups using homogenised right lung lobes, brain, liver, and spleen tissues (Figure 6m–o). No viruses were recovered from liver and spleen plaque assays (data not shown). Viral loads were similar between non-treatment groups in all three variants with geometric means of 7.76 × 10^4^, 4.77 × 10^4^, and 1.13 × 10^4^ PFU/organ for variant L, Alpha, and Beta respectively. Viral titres did not show a decrease after calcitriol treatment for L and Beta variants with levels determined to be at 5.31 × 10^4^ and 7.83 × 10^3^ PFU/organ respectively. Lung titres in Alpha variant infected mice were significantly different in the treatment group with a quantified load of 1.6 × 10^5^ PFU/organ. Virus load in brain tissues were highly variable regardless of variant or treatment statuses. Titrated levels of virus load for non-treatment groups were 3.28 × 10^4^, 2.6 × 10^6^, and 2.17 × 10^4^ PFU/organ and for treatment groups, 2.98 × 10^5^, 2.24 × 10^5^, 3.36 × 10^2^ PFU/organ for variant L, Alpha, and Beta respectively.

## 4. Discussion

The use of Host-directed therapies (HDTs) for the prevention of infections is certainly not a new idea. Most of these therapies however rely mainly on the use of vaccines, convalescent plasma, and monoclonal antibodies [28,29]. Small molecule HDTs have been used adjunctively for diseases such as tuberculosis [30] and have been proposed for viral pandemics [31]. This strategy would overcome some of the costs and challenges associated with antiviral production, including the emergence of drug resistance [32]. Here, vitamin D is a well-known modulator of host immune responses through the production of antimicrobial peptides such as cathelicidin to promote autophagy [33]. It has proven essential for host defenses against many intracellular pathogens including respiratory pathogens such as *Mycobacterium tuberculosis*, and has been shown to also possess anti-inflammatory properties [33]. A recent study by Smith and colleagues [15] showed an association between vitamin D deficiency, SARS-CoV-2 infection, and COVID-19-associated mortality. The authors speculated that vitamin D supplementation could protect against SARS-CoV-2 infection and improve patient disease outcomes [15], and our finding certainly provides credence to this hypothesis. Given that calcitriol-mediated inhibition occurred upon post-treatment of Vero E6 cells and hNECs, it is likely that its mechanism of antiviral action targets the post-entry phase of viral replication. Furthermore, we also confirm that SARS-CoV-2 is involved in the VDR pathway through tracking the changes in mRNA expression of VDR, 24(OH)ase, and LL-37 across time and calcitriol treatment. Our results showed that SARS-CoV-2 could alter the vitamin D metabolic pathway and limit the functionality of endogenous calcitriol in Vero E6 and Huh7 cells, although the direction of effect for VDR and 24(OH)ase can be different between cell lines. Despite this, when exogenous calcitriol is added to both cell lines, calcitriol upregulates the genes important in controlling virus replication, such as 24(OH)ase and LL-37. Specifically, the upregulation of LL-37 is of interest due to its role as a selective cathepsin L inhibitor [18].

To mediate entry into host cells, SARS-CoV-2 relies on host proteases such as furin, transmembrane protease serine 2 (TMPRSS2), and host peptidases to act as receptors for virus attachment and membrane fusion with the target cell membrane [34]. Here, host peptidase cathepsin L plays an important role to cleave the virus spike protein and enhance virus entry into host cells [35]. Since LL-37 has been shown to be a selective cathepsin L inhibitor [18], it is likely that the presence of LL-37 might reduce the ability of SARS-CoV-2 to infect cells. This is supported by a recent study by Wang and co-workers [36], where they showed that LL-37 serves a dual function in inhibiting SARS-CoV-2 infection by binding to the receptor binding domain of S1 and decreasing the recruitment of ACE2 in a process known as spike blocking and ACE2 cloaking. Hence, it is likely that calcitriol, when taken into the cells, activates the VDR pathway to upregulate LL-37. While primary SARS-CoV-2 infection will continue to occur as calcitriol targets the post-entry steps of infection, the upregulation of LL-37 via the VDR pathway will subsequently prevent secondary SARS-CoV-2 infection among cells, resulting in an overall reduction of virus titre. However, additional studies, such as testing whether silencing expression of the gene encoding LL-37 attenuates calcitriol-induced antiviral activity, will need to be performed to further dissect the antiviral mechanism of calcitriol.

While calcitriol displayed significant in vitro efficacy, the same was not reflected in our in vivo studies. K18-hACE2 mice pre- and post-treated with calcitriol failed to exhibit any protective effects when challenged with wild-type SARS-CoV-2 or its variants. There could be a few reasons why it was not effective in mice. Firstly, mice may be utilizing vitamin D in a different way as the species used for the cell-based studies and secondly, LL-37 is not vitamin D-regulated in mice [37]. The calcitriol dose given to mice may also be too low to keep levels sufficient [25]. Since the mouse model is unable to recapitulate the in vitro findings of this study, the same study should be repeated in another suitable animal model (such as ferrets, hamsters, or non-human primates) to corroborate the results of the cell-based studies. Given that the calcitriol concentration administered to the mice was translated to the human equivalent dosage of between 0.5 to 5 mcg/kg, the results generated from our in vivo studies suggested that calcitriol treatment may not help to prevent SARS-CoV-2 infection or improve patient disease outcomes at its usual therapeutic dosage. Additionally, if patients were treated with higher concentrations of calcitriol e.g., hyperparathyroidism patients, hypercalcemia may develop as a common side effect. Hence, a more in-depth pharmacokinetic study should also be performed before calcitriol can be repurposed for COVID-19 prophylaxis or treatment.

## Figures and Tables

**Table 1:** Primary hits selected for further validation against SARS-CoV-2 infection.

Pharmaceutical Class	No. of Compounds with Activity against SARS-CoV-2			Total No. of Compounds for Each Class
	FDA-Approved Drugs	ACE2 Inhibitors	Flavonoids	
Antiviral agents	3	2		5
Antibacterial agents	9	4		13
Antifungal agents	3			3
Antiparasitic agents	2			2
Anticancer agents	3	3		6
Antihistamines	5			5
Antihypertensives	1	1		2
Antiarrhythmic		1		1
Anticoagulants		1		1
Antispasmodic	1			1
Proton pump inhibitors	2			2
Ion channel blockers	3			3
Non-steroidal anti-inflammatory drugs	3			3
Anti-inflammatory agents	2	4		6
Phosphodiesterase inhibitors	1			1
Lipid, sterol metabolism inhibitors	1	1		2
Signalling kinase inhibitors	2	2		4
Neurotransmitter inhibitors	3			3
Nucleic acid synthesis inhibitors	2			2
Histone deacetylase inhibitors		1		1
Flavonoids		1	34	35
Others	10	10		20
Total	56	31	34	121

**Table 2:** Summary of compounds identified from the primary screen that exhibit potential antiviral effects against SARS-CoV-2 infection.

Compound	Chemical Structure	Pharmaceutical Class	Known Mechanism	Indication	FDA-Approved
Citicoline	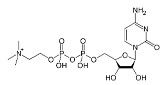	Others	Intermediate in the synthesis of Phospatidylcholine	Proposed for use in traumatic brain injuries, stroke and vascular dementia due to potential neuroprotective effects	No
Pravastatin Sodium	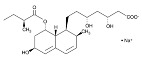	Lipid, sterol metabolism Inhibitor	Inhibits HMG-CoA reductase, thus inhibiting the synthesis of cholesterol	Prevention of cardiovascular disease and treatment of hypercholesterolemia	Yes
Tenofovir Alafenamide	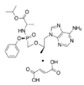	Antiviral	Nucleoside inhibitor of viral reverse transcriptase	Prodrug form of tenofovir, used for treatment of HIV and chronic Hepatitis B infections	Yes
Imatinib Mesylate	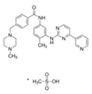	Signalling kinase inhibitor	Inhibits Bcr-Abl tyrosine kinase	Treatment of chronic myelogenous leukemia, gastrointestinal stromal tumors and various other cancers	Yes
Calcitriol	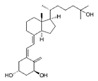	Others	Active metabolite of vitamin D_3_	Treatment of secondary hyperparathyroidism and metabolic bone disease, hypocalcemia, osteoporosis	Yes
Dexlansoprazole	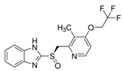	Proton pump Inhibitor	Inhibits H^+^/K^+^ ATPase, resulting in decreased secretion of HCl into the gastric lumen	Treatment and management of gastroesophageal reflux, erosive esophagitis	Yes
Prochlorperazine Dimaleate	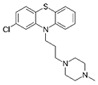	Neurotransmitter inhibitor	Blocks D_2_ dopamine receptors in the brain	Treatment of severe nausea and vomiting, as well as short-term management of anxiety and schizophrenia	Yes

**Table 3:** CC_50_, IC_50_, and SI values observed from validation of primary hits in Vero E6 and Huh7 cells.

Compound	CC_50_ (μM)	IC_50_ (μM)	Selectivity Index
Vero E6 Cells			
Citicoline	>20	-	-
Pravastatin Sodium	>20	<0.01	>2000
Tenofovir Alafenamide	>20	-	-
Imatinib Mesylate	>20	<0.01	>2000
Calcitriol	>20	<0.01	>2000
Dexlansoprazole	>20	<0.01	>2000
Prochlorperazine Dimaleate	>20	-	-
HuH7 Cells			
Citicoline	>20	-	-
Pravastatin Sodium	>20	-	-
Tenofovir Alafenamide	>20	-	-
Imatinib Mesylate	>20	-	-
Calcitriol	4.692	-	-
Dexlansoprazole	>20	2.479	>8.0678
Prochlorperazine Dimaleate	15.73	-	-

Note: CC_50_ and IC_50_ values were derived from data obtained from cell viability and dose-dependent inhibition studies respectively.

## Data Availability

All data is available in the main text or the Supplementary Materials.

## Supplementary Materials

The following supporting information can be downloaded at: https://www.mdpi.com/article/10.3390/pharmaceutics15030925/s1, Figure S1: Full length western blots of SARS-CoV-2 spike and VDR proteins in Vero E6 cells; Figure S2: Physiological scoring of K18-hACE2 mice treated by calcitriol; Figure S3: Lung biopsy of K18-hACE2 mice treated by calcitriol; Figure S4: In vivo calcitriol treatment by oral gavage; Table S1: List of primary hits identified from the primary screen performed using an FDA-approved library; Table S2: List of primary hits identified from the primary screen performed using an ACE2 inhibitors library; Table S3: List of primary hits identified from the primary screen performed using a flavonoid library.
