# Supplementary material for: Evaluation of In Vitro and In Vivo Antiviral Activities of Vitamin D for SARS-CoV-2 and Variants
Source: Pharmaceutics. 2023 Mar 12;15(3):925. doi: 10.3390/pharmaceutics15030925 (PMC10058714; doi:10.3390/pharmaceutics15030925)
Supplement: Supplementary file 1 [file pharmaceutics-15-00925-s001.zip › pharmaceutics-2125262-supplementary.pdf]

Article

Supplementary Materials: Evaluation of In Vitro and In Vivo Antiviral Activities of Vitamin D for SARS-CoV-2 and Variants

Chee-Keng Mok , Yan Ling Ng, Bintou Ahmadou Ahidjo , Zhen Qin Aw, Huixin Chen, Yi Hao Wong , Regina Ching Hua Lee, Marcus Wing Choy Loe, Jing Liu, Kai Sen Tan, Parveen Kaur, De Yun Wang , Erwei Hao, Xiaotao Hou, Yong Wah Tan, Jiagang Deng and Justin Jang Hann Chu

Figure S1. Full length western blots of SARS-CoV-2 spike and VDR proteins in Vero E6 cells.

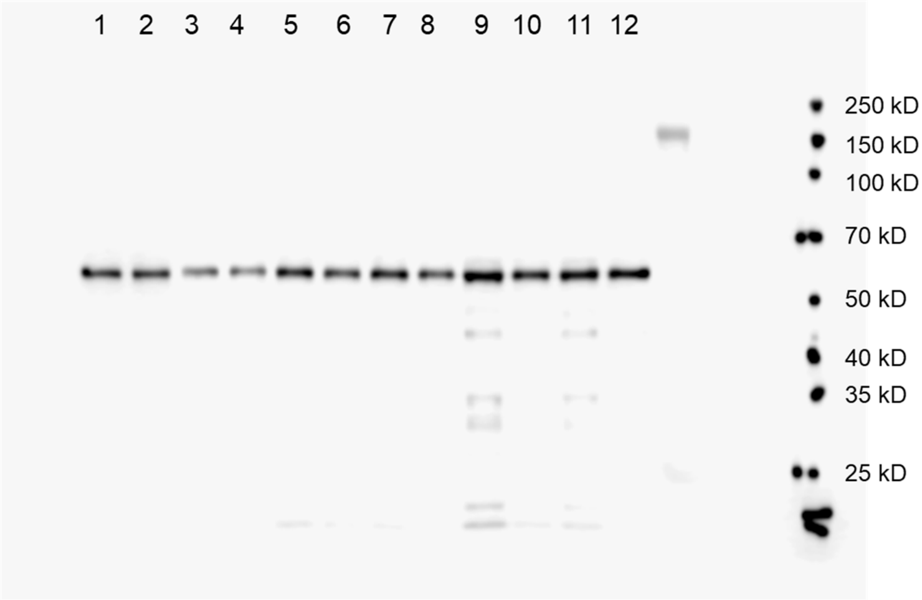

α-vitamin D receptor (1:3000 in 5% BSA)
Proteintech α-Vitamin D Receptor (Cat No.: 16922-1-AP)

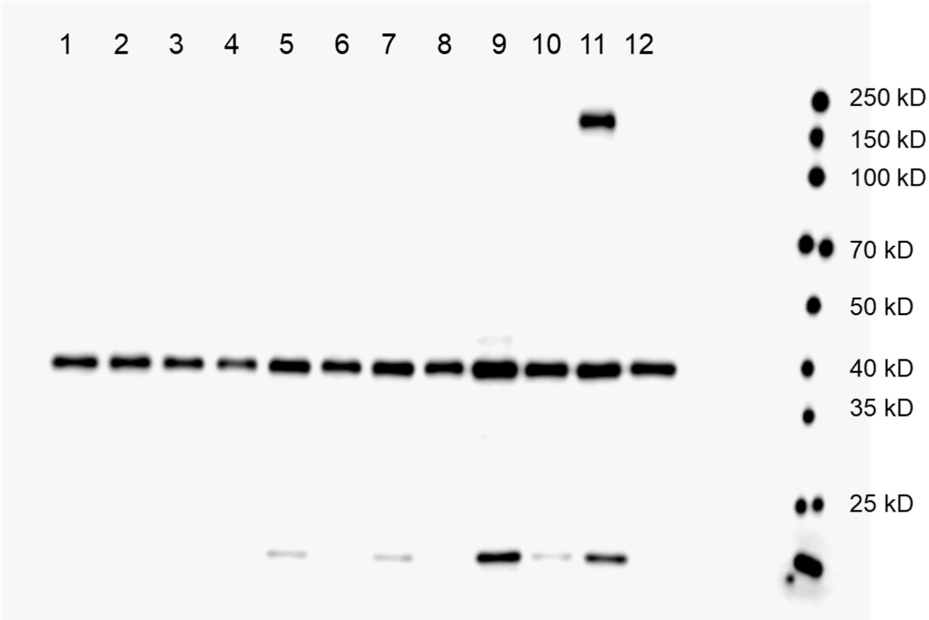

α-beta actin (1:10000 in 5% BSA)

α-SARS-CoV-2 S1 RBD (1:1500 in 5% BSA)
Sinobiological α-SARS-CoV-2 S1/RBD (Cat No.: 40592-T62)

Figure S2. Physiological scoring of K18-hACE2 mice treated by calcitriol.

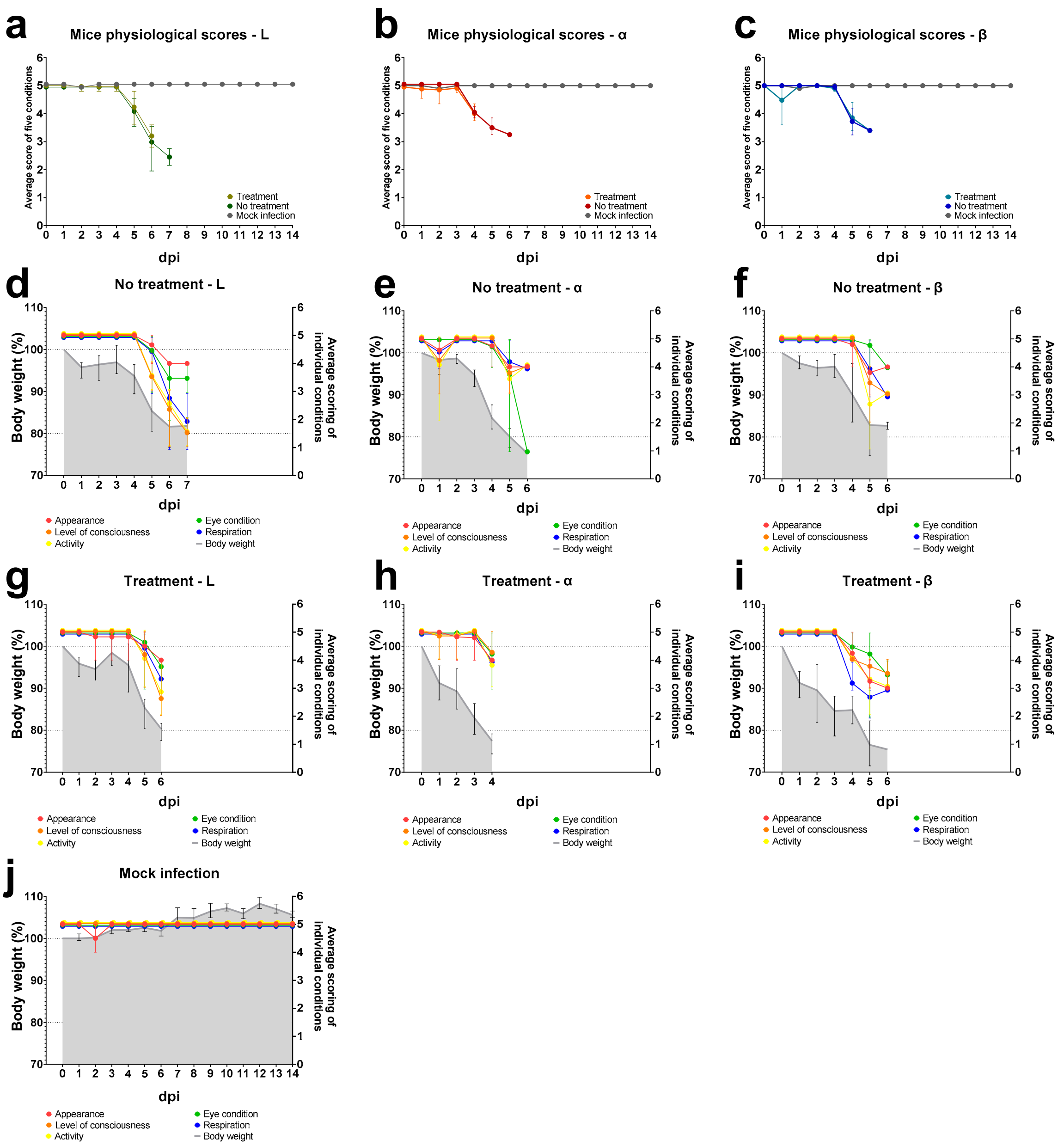

Average score of the five physiological conditions between treatment and no treatment groups over the course of the infection for all three variant infected mice (**A, B, C**). Scoring is based on the average of five physiological criteria: appearance, level of consciousness, activity, eye condition, and respiration, and scored from 1 (worst) to 5 (normal) of each group. Breakdown of individual physiological criteria score against average body weight loss of each treatment group by variants (**D-J**).

Figure S3. Lung biopsy of K18-hACE2 mice treated by calcitriol.

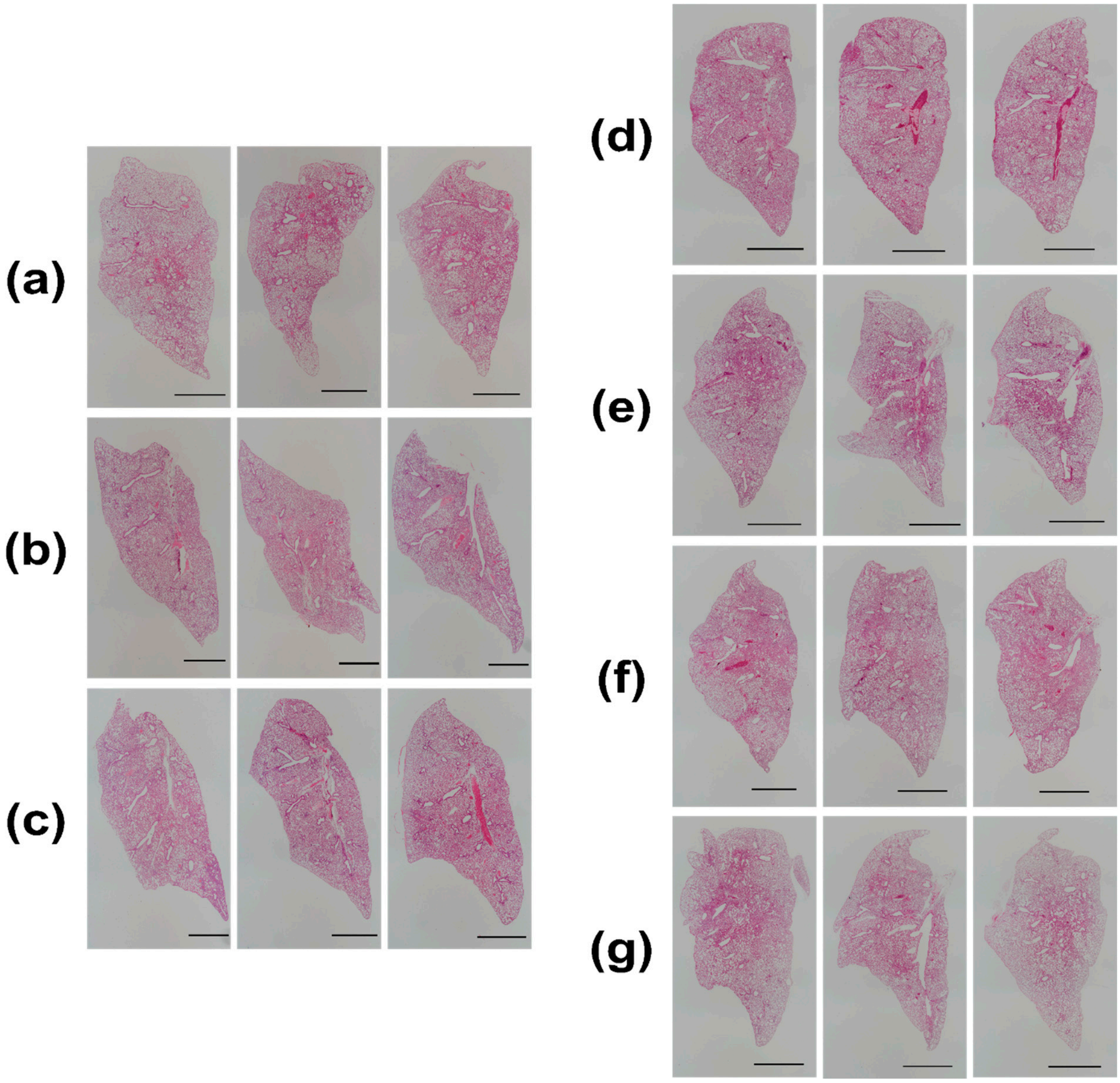

H&E staining of 4dpi mice's lung biopsy from (**A**) mock treatment group; no treatment group infected with the (**B**) wild-type, (**D**) alpha, (**F**) beta variants of SARS-CoV-2; and treatment group infected with the (**C**) wild-type, (**E**) alpha, (**G**) beta variants of SARS-CoV-2. (Magnification: 5x, scale bars: 2000µm)

Figure S4. ***In vivo*** **calcitriol treatment by oral gavage.**

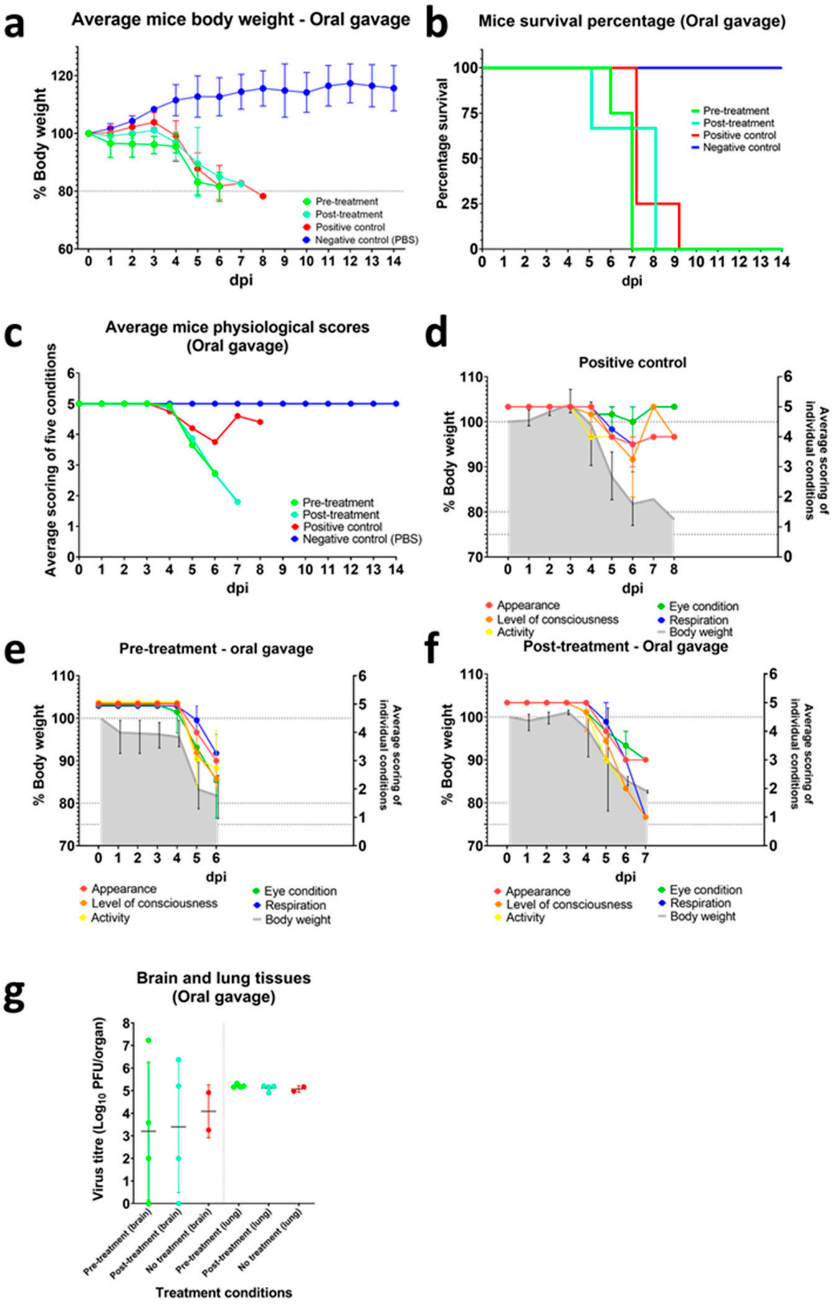

Low dose of calcitriol (0.5 ug/kg) have been given by oral gavage to pre-treatment and post-treatment groups. Positive and negative controls were no treatment and given PBS. Positive, pre- and post-treatment groups were infected by wild-type SARS-CoV-2 (10^3 PFU) after 7-days of pre-treatment. **(A)** Body weight loss, **(B)** survival rate, **(C)** average of physiological scoring, **(D)** individual physiological criteria of positive control, **(E)** pre-treatment, and **(F)** post-treatment groups were recorded. **(G)** Viral load of lung and brain after 4 dpi were examined by plaque assay.

Table S1. List of primary hits identified from the primary screen performed using an FDA-approved library.

Compound/Treatment	Image	Compound/Treatment	Image
Uninfected	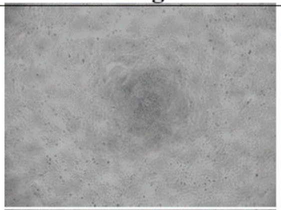	0.1% DMSO	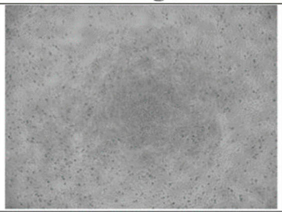
Remdesivir	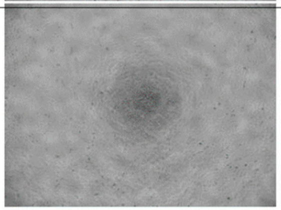		
Ramelteon (TAK-375)	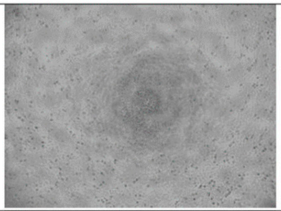	Telmisartan	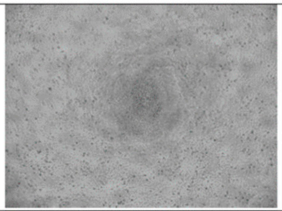
Masitinib	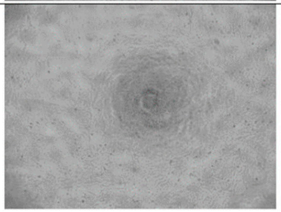	Rifampin	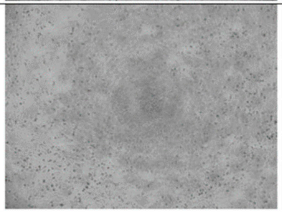

Compound/Treatment	Image	Compound/Treatment	Image
MDV3100 (Enzalutamide)	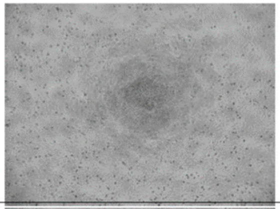	Amorolfine Hydrochloride	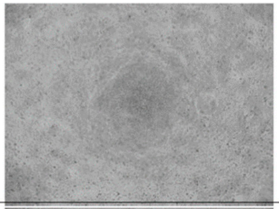
Everolimus	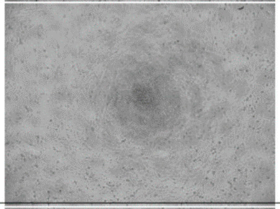	Manidipine	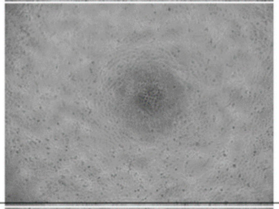
Fulvestrant (Faslodex)	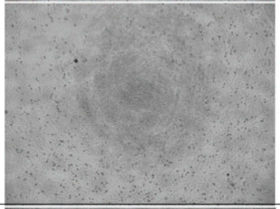	Clomifene Citrate	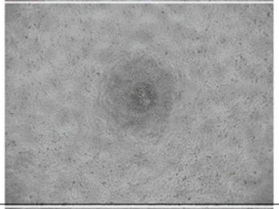
Imatinib Mesylate	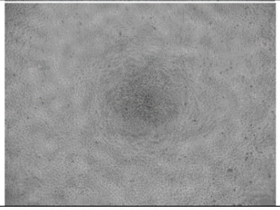	Econazole Nitrate	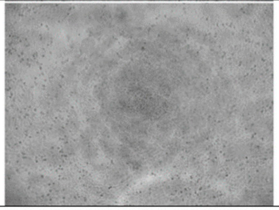

Compound/Treatment	Image	Compound/Treatment	Image
Mianserin Hydrochloride	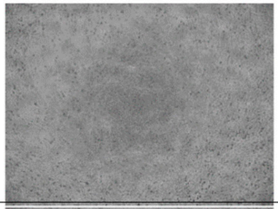	Phenacetin	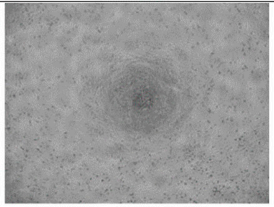
Trilostane	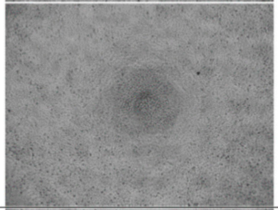	Lonidamine	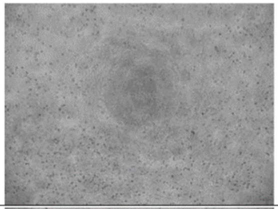
Mercaptopurine	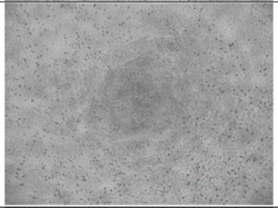	Idebenone	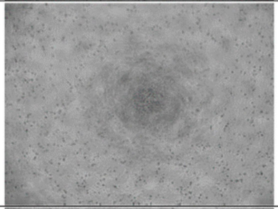
Artemisinin	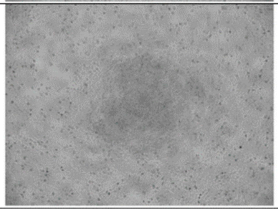	Hygromycin B	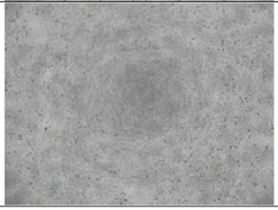

Compound/Treatment	Image	Compound/Treatment	Image
Cetirizine Dihydrochloride	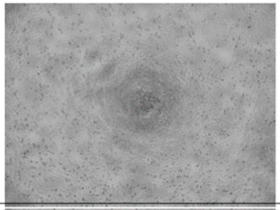	Alverine Citrate	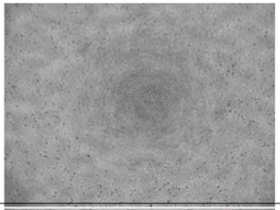
Loratadine	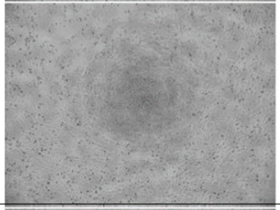	Doxycycline Hydrochloride	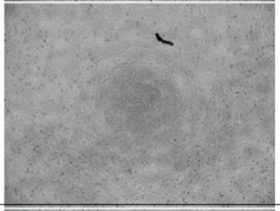
Ketoconazole	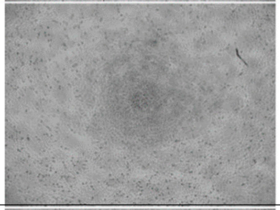	Creatinine	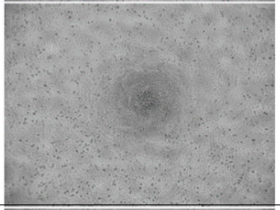
Lopinavir	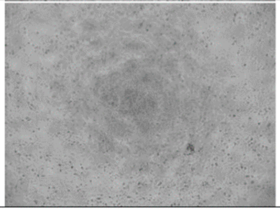	Benztropine Mesylate	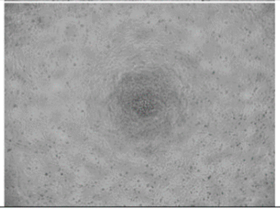

Compound/Treatment	Image	Compound/Treatment	Image
Ranolazine Dihydrochloride	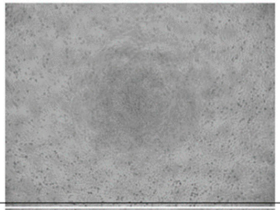	Clinafloxacin	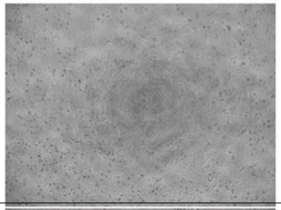
Tranilast	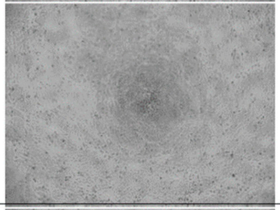	Altrenogest	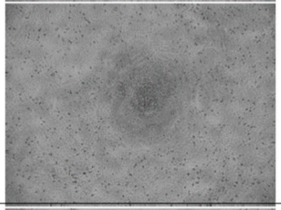
Ofloxacin (Floxin)	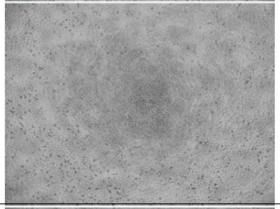	Avanafil	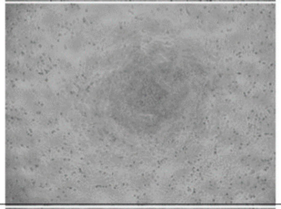
Erythromycin	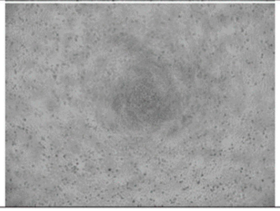	Trifluoperazine Dihydrochloride	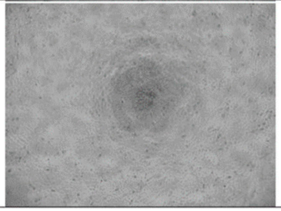

**Compound/Treatment**	**Image**	**Compound/Treatment**	**Image**
Moxifloxacin Hydrochloride	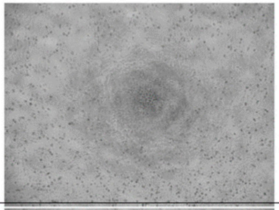	Sertraline Hydrochloride	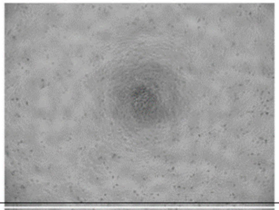
Calcitriol	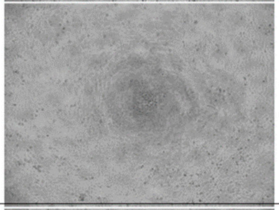	Dexlansoprazole	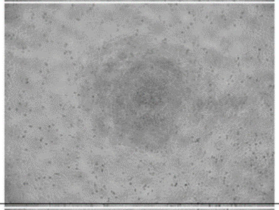
Telaprevir	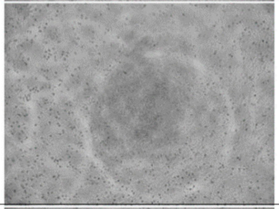	Closantel Sodium	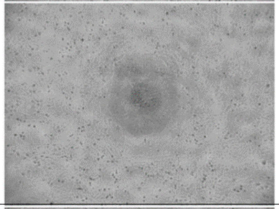
Calcifediol	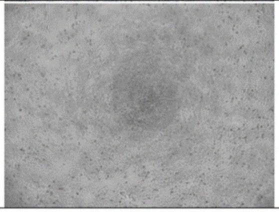	Famprofazone	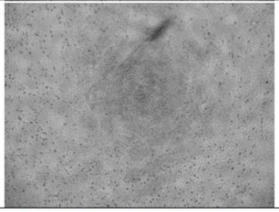

Compound/Treatment	Image	Compound/Treatment	Image
Cyclosporine	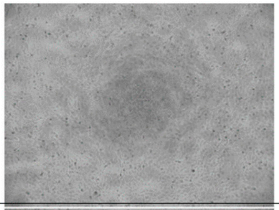	Metaproterenol Sulfate (Orciprenaline)	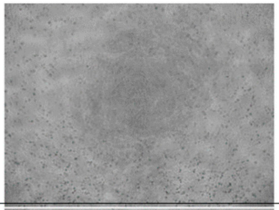
Telbivudine	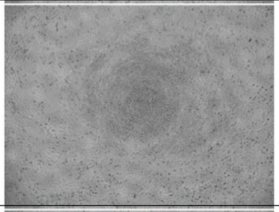	Ractopamine Hydrochloride	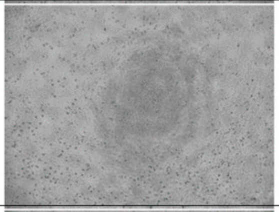
Nevirapine	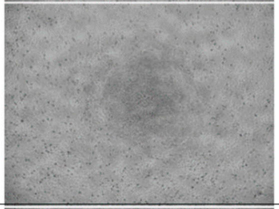	Terfenadine	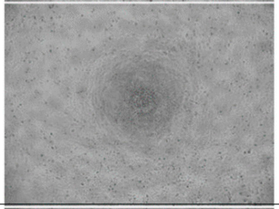
Esomeprazole	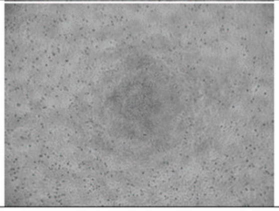	Thiostrepton	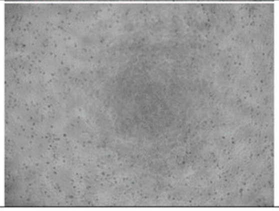

Compound/Treatment	Image	Compound/Treatment	Image
Progesterone	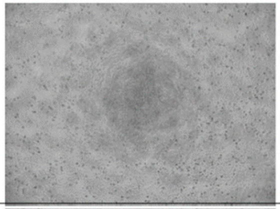	Glafenine Hydrochloride	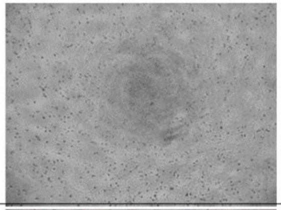
Nimodipine	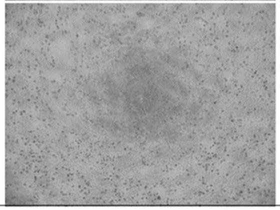	Meclocycline Sulfosalicylate	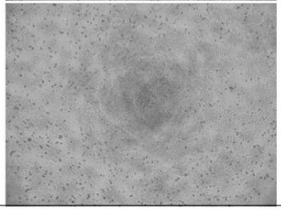

Table S2. List of primary hits identified from the primary screen performed using an ACE2 inhibitors library.

Compound/Treatment	Image	Compound/Treatment	Image
Uninfected	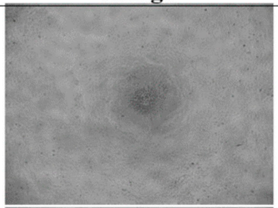	0.1% DMSO	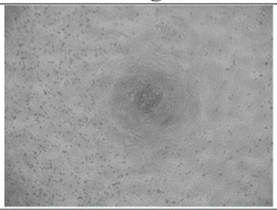
Remdesivir	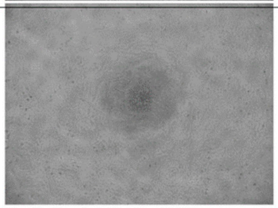		
Citicoline	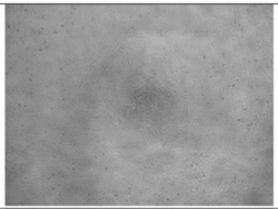	Nuezhenide	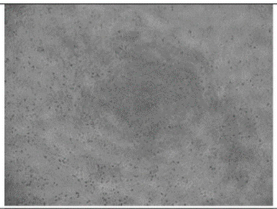
Cefpiramide Acid	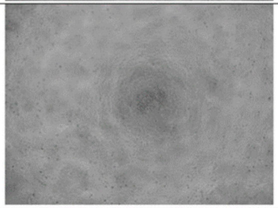	Tepotinib	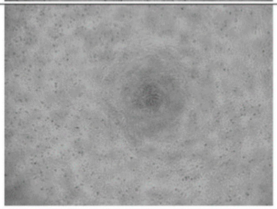

Compound/Treatment	Image	Compound/Treatment	Image
Calcium Follinate	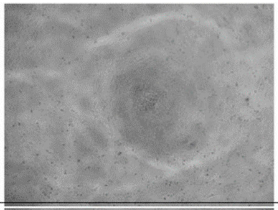	Mulberroside A	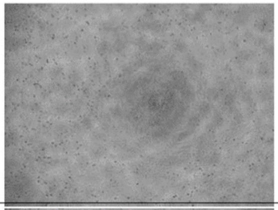
Dronedarone Hydrochloride	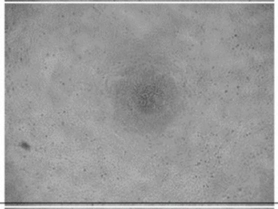	4,5-Dicaffeoylquinic acid	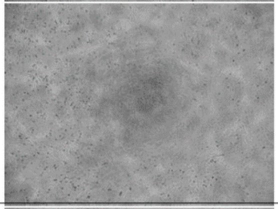
Pravastatin Sodium	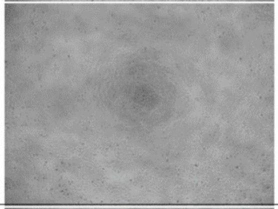	Crocin I	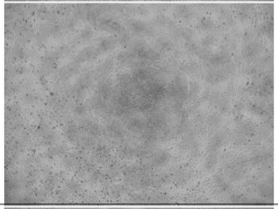
Tenofovir Alafenamide	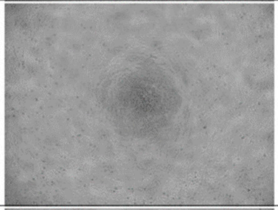	PLX8394	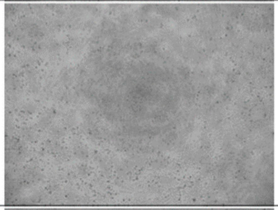
Navitoclax	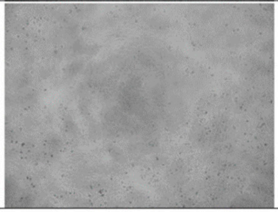	Complanatoside	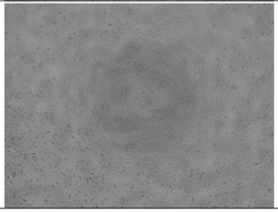

**Compound/Treatment**	**Image**	**Compound/Treatment**	**Image**
Bimatoprost	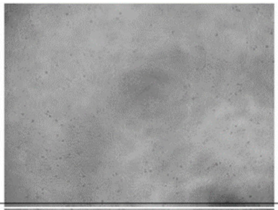	1,2-Dipalmitoyl-sn-glycerol	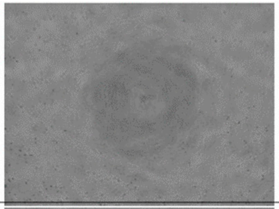
Echinacoside	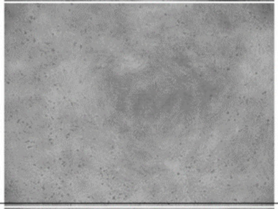	rac-Glycerol 1-myristate	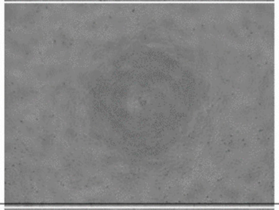
CUDC101	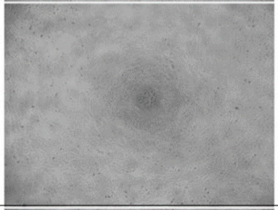	Cefotiam Hexetil Hydrochloride	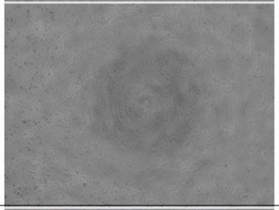
PHT427	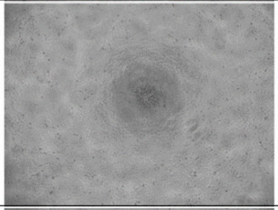	Dabigatran Etexilate Mesylate	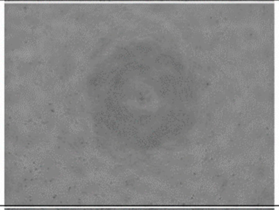
Puromycin Dihydrochloride	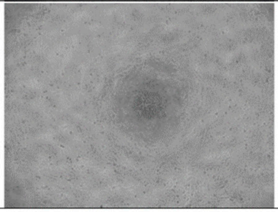	Glycerol Tridecanoate	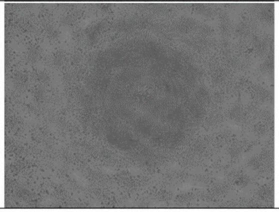

Compound/Treatment	Image	Compound/Treatment	Image
UNC0638	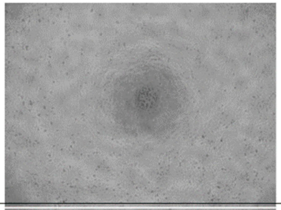	ARV-825	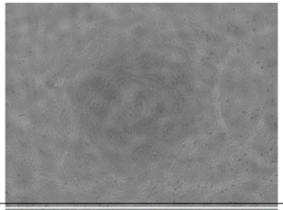
Homoharringtonine	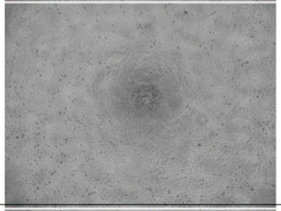	Levobetaxolol Hydrochloride	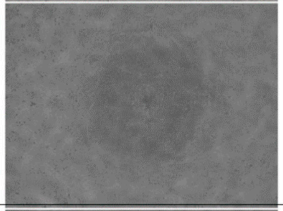
Calpeptin	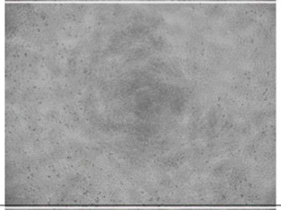	Travoprost	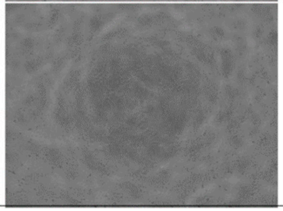
Crocin II	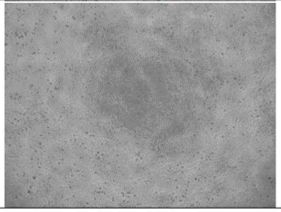		

Table S3. List of primary hits identified from the primary screen performed using a flavonoid library.

Compound/Treatment	Image	Compound/Treatment	Image
Uninfected	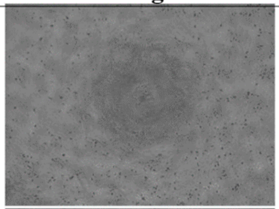	0.1% DMSO	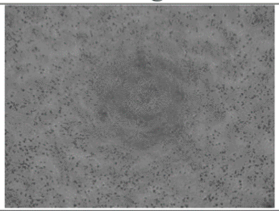
Remdesivir	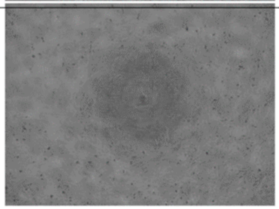		
ST002047	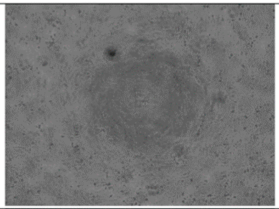	ST079547	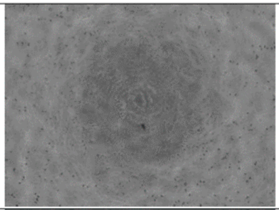
ST014848	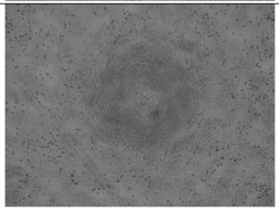	ST085666	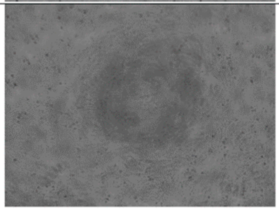

ST055995	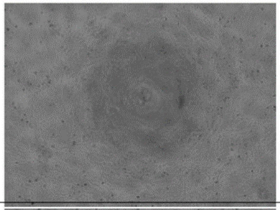	ST076522	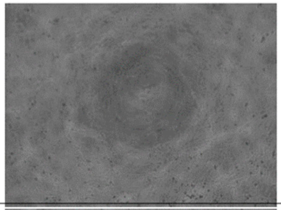
ST056004	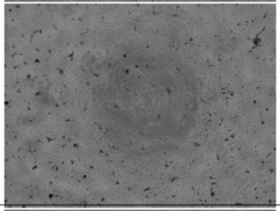	ST083654	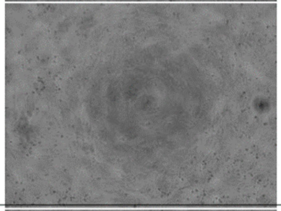
ST056249	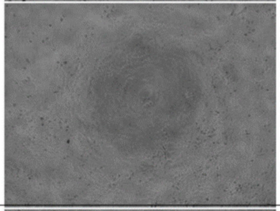	ST081389	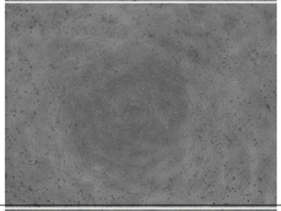
ST056248	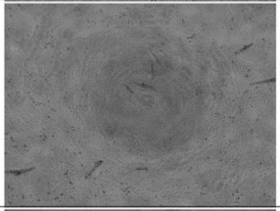	ST076533	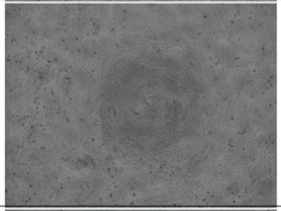
ST057711	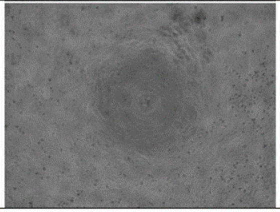	ST095414	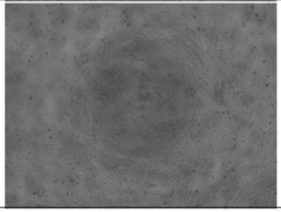

ST058442	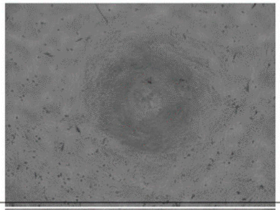	ST097097	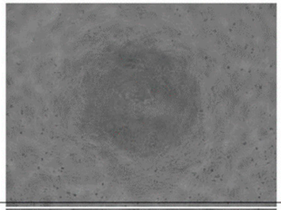
ST057646	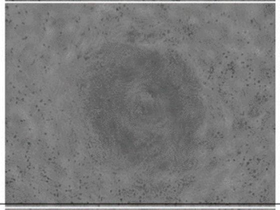	ST094884	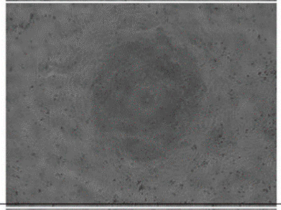
ST059590	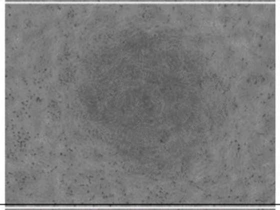	ST095197	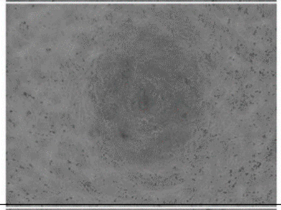
ST069324	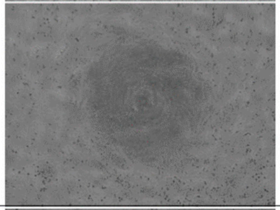	ST095849	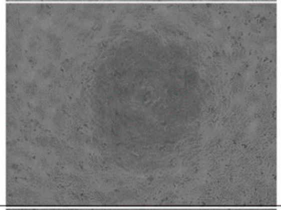
ST075446	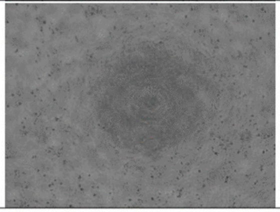	ST097570	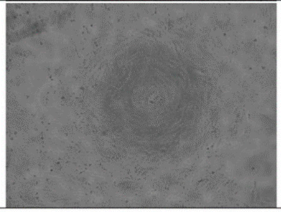

ST076534	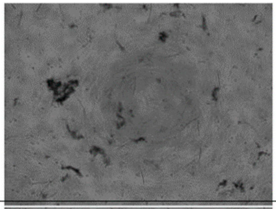	ST020733	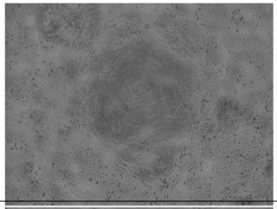
ST079949	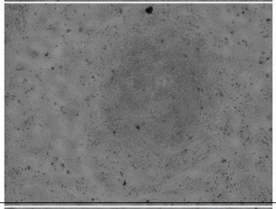	ST074501	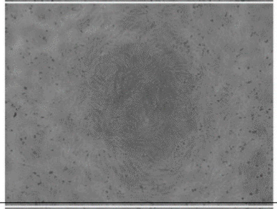
ST083092	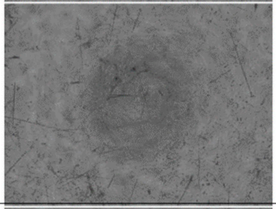	ST074530	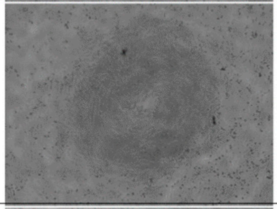
ST083658	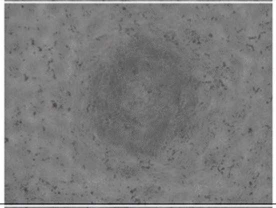	ST077114	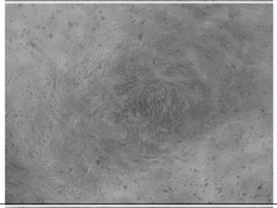
ST077135	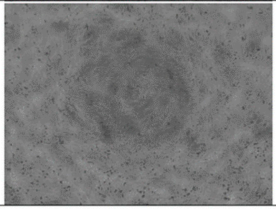	ST077122	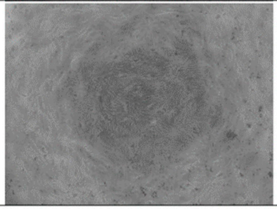
